# Genomic copy-number variants drive apoptotic evasion underlying acquired resistance to immune checkpoint inhibitors

**DOI:** 10.1016/j.immuni.2025.10.001

**Published:** 2025-10-31

**Authors:** Mingming Wu, Shiyue Yang, Zhentao Yang, Jian Fan, Shirley H. Lomeli, Prashanthi Dharanipragada, Gatien Moriceau, Robert Damoiseaux, Mark C. Kelley, Carlos N. Prieto-Granada, Alessio Giubellino, Mehdi Nosrati, Mohammed Kashani-Sabet, Kevin B. Kim, Douglas B. Johnson, Sixue Liu, Roger S. Lo

**Affiliations:** 1 Division of Dermatology, Department of Medicine, David Geffen School of Medicine, University of California, Los Angeles, Los Angeles, CA, USA; 2 Department of Molecular and Medical Pharmacology, David Geffen School of Medicine, University of California, Los Angeles, Los Angeles, CA, USA; 3 Jonsson Comprehensive Cancer Center, David Geffen School of Medicine, University of California, Los Angeles, Los Angeles, CA, USA; 4 Department of Bioengineering, University of California, Los Angeles, Los Angeles, CA, USA; 5 California NanoSystems Institute, University of California, Los Angeles, Los Angeles, CA, USA; 6 Department of General Surgery, Vanderbilt University Medical Center, Nashville, TN, USA; 7 Vanderbilt Ingram Cancer Center, Vanderbilt University Medical Center, Nashville, TN, USA; 8 Department of Pathology, Vanderbilt University Medical Center, Nashville, TN, USA; 9 Department of Laboratory Medicine and Pathology, University of Minnesota, Minneapolis, MN, USA; 10 Masonic Cancer Center, University of Minnesota, Minneapolis, MN, USA; 11 Center for Melanoma Research and Treatment, California Pacific Medical Center, San Francisco, CA, USA; 12 California Pacific Medical Center Research Institute, San Francisco, CA, USA; 13 Division of Hematology/Oncology, Department of Medicine, Vanderbilt University Medical Center, Nashville, TN, USA

## Abstract

Patients who initially respond to immune checkpoint inhibitors (ICIs) often relapse. Here, we studied how disease-progressive (DP) clinical melanomas evolve genomically to acquire ICI resistance. Compared to patient-matched pretreatment tumors, DP tumors recurrently amplified and/or deleted anti-apoptotic and/or pro-apoptotic genes, respectively. By chronic exposure to killer T cells or ICI therapy, we derived acquired-resistant (AR) human melanoma cell lines and murine melanoma tumors that recapitulate co-occurrent copy-number variants (CNVs) of apoptotic genes observed in DP melanomas. AR and DP subclones expanded shared, private, and, in some subclones, preexistent driver CNVs. Compared to isogenic parental cells, AR melanoma cells attenuated apoptotic priming but, with overexpression of deleted pro-apoptotic genes, recovered mitochondrial priming and sensitivity to killer T cells or ICIs. In mice, pharmacologically reducing the apoptotic threshold of ICI persisters prevented relapses. Thus, CNVs can drive the evolution of resistance to ICIs in melanoma, with tumor cell-intrinsic apoptotic threshold representing a target to curtail persister evolution.

## In brief

Cancers initially responsive to immune checkpoint inhibitors (ICIs) often relapse, and localized cancers can recur after surgery plus adjuvant ICI therapy. Wu et al. find that relapsing and recurrent melanomas evolve permutations of DNA copy-number variants in genes that confer resistance to T cell killing. Pharmacologically priming persister cancer cells to apoptosis may thwart resistance evolution.

## Graphical Abstract

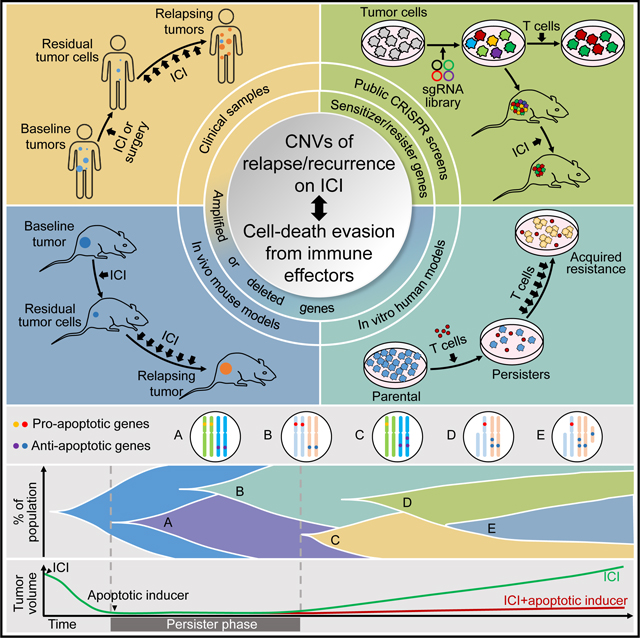

## INTRODUCTION

Resistance to immune checkpoint inhibitors (ICIs) limits their cancer-curative potential.^1–5^ In primary resistance, patients do not respond to ICIs from the outset of treatment. Adaptive resistance may manifest as transient objective responses or disease stabilization, providing a bridge to acquired resistance (AR). In AR, tumor relapses can sometimes follow initial responses by years. In cutaneous melanoma, disease progression (DP) after prolonged objective responses to ICIs develops in 20%–60% of patients.^6^ Notably, knowledge of the clinical mechanisms of AR lags behind that of innate or adaptive resistance, despite the widespread application of ICIs in cancer treatment.

Tumor-cell-intrinsic and -extrinsic factors contribute to innate and adaptive resistance. The former category includes dysregulated PTEN^7^, WNT,^8^ and interferon (IFN)^9,10^ pathways as well as low neoantigen burden^11,12^ and defective antigen presentation.^13^ For example, bi-allelic, loss-of-function mutations in *B2M* (in the antigen-presentation pathway) result from a hemizygous deletion plus a small insertion-and-deletion mutation, the latter of which suggests defective DNA mismatch repair as a resistance-driving force.^13^ However, the mutational versus non-genomic basis of intrinsic factors remains largely unclear. An extensive literature^14^ implicates tumor-cell-extrinsic innate and/or adaptive resistance factors involving various immune cell types (e.g., regulatory T cells and M2 macrophages) and immune checkpoints distinct from PD-1 and CTLA-4. Moreover, a signature encompassing both tumor-cell-intrinsic and -extrinsic (e.g., mesenchymal, angiogenic, and wound healing) processes is associated with innate anti-PD-1 resistance.^11^

The evolution of AR to ICIs in melanoma^15^ and other cancers^6^ results in somatic non-synonymous mutations in the IFN receptor signaling and antigen-presentation pathways. Transcriptomic analysis of non-small cell lung cancer shows that AR to anti-PD-(L)1 associates with persistent CD8^+^ T cell inflammation but a dysfunctional IFN response.^16^ Both small-scale mutational and large-scale chromosomal instability processes contribute to the evolution of AR in cancer. To date, our knowledge of the contributions of large-scale genomic instability, including somatic copy-number variants (CNVs), to AR on ICIs is limited. Mechanisms of evolvability may inform therapeutic targets to prevent relapses.^17–19^

Comprehensive CRISPR-Cas9 screens have identified genes that modulate killing of tumor cells by T cells.^20–42^ However, it is common for such experimental screens in various cancer models to be performed over time scales that are much shorter than the evolutionary durations underlying clinical AR. Thus, whether the results or hits of such functional screens can faithfully inform mechanisms of clinical relapses on ICIs remains unknown.

Here, using whole-exome sequences (WESs) from pre and post melanomas and patient-matched normal tissues, we identified and functionalized somatic, recurrent mutations specific to relapsing or recurrent melanomas after anti-PD-1 ± anti-CTLA-4 therapy. Because of melanomas’ high CNV burden, we focused on recurring and DP-specific amplified or deleted genes that CRISPR-Cas9 screens have identified as T cell sensitizer or resister genes, respectively. This integrative analysis revealed co-occurring and DP-specific apoptosis-downregulatory CNVs. Coculturing human melanoma cells with human leukocyte antigen (HLA)-/antigen-specific killer T cells and treating murine melanoma tumors with ICIs generated AR models that enabled somatic genomic analysis. Single-cell whole-genome sequence (scWGS) analysis of AR models and clinical tumors revealed pre-existent and subclone-private AR CNVs that converged on apoptotic genes, known innate ICI resistance genes, and gene hits from published CRISPR-Cas9 screens. Finally, using AR models, we showed that restoring apoptotic signaling rescued the CNV-based apoptotic defects of AR and that pharmacologic induction of mitochondrial priming in ICI persisters selected against ICI relapses. These findings represent an evolutionary model in which CNV genomic instability engenders root-plus-branch mechanisms of relapses on ICI therapy.

## RESULTS

### Recurrent DP-specific CNVs attenuate tumor-cell death

**Figure 1.**
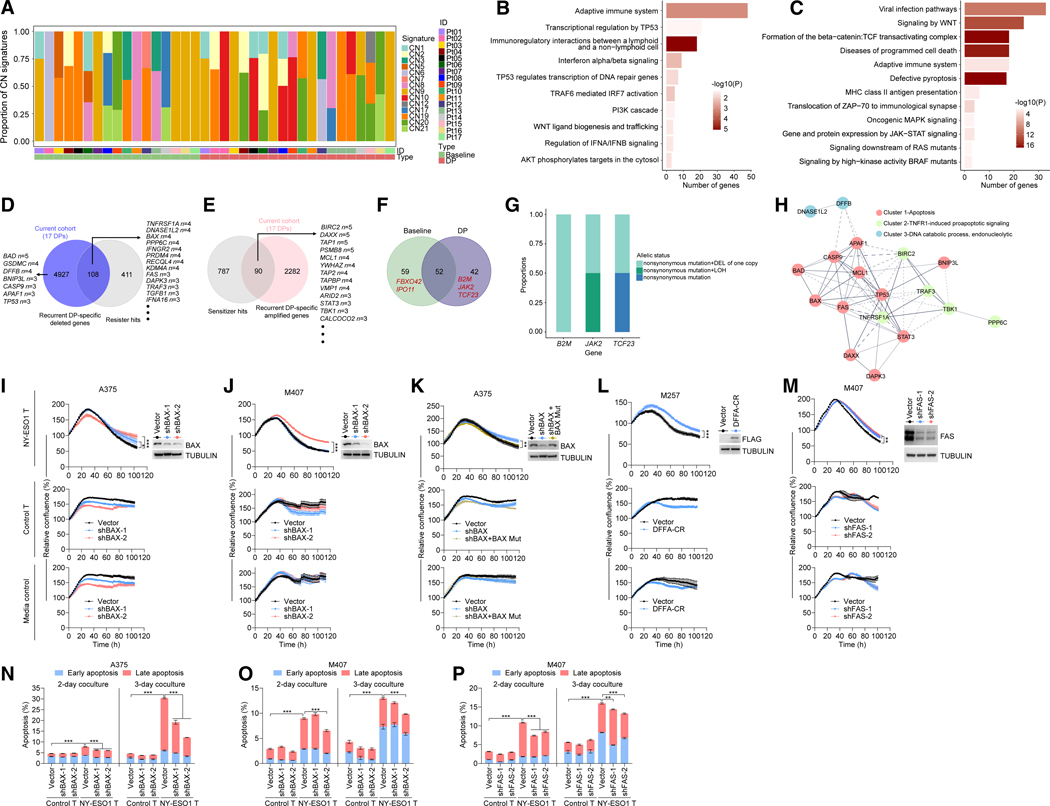
Integrative genomic analysis to functionalize relapse-specific alterations identifies CNVs of apoptotic genes (A) Attribution frequencies of CN signatures in patient-matched baseline and DP CNVs. (B and C) Significantly enriched immune-, cell death-, and innate ICI resistance-related Reactome gene sets of DP-specific (patient-matched), recurrently (≥4 of 17 patients) deleted (B) or amplified (C) genes. *p* values computed using the hypergeometric test. (D) The numbers of recurrent (≥3 of 17 patients), DP-specific (patient-matched)-deleted genes (left circle), resister genes from CRISPR-Cas9 functional screens (right circle), and overlapping genes (select genes shown with the numbers of patients affected). (E) As in (D), except for amplified and sensitizer genes. (F) The numbers of baseline (left circle), DP (right circle), and overlapping provisional SMGs. SMGs identified as sensitizer (left) or resister (right) genes highlighted in red. (G) Allelic status (CN and non-synonymous mutations) of DP-specific SMGs (*B2M*, *JAK2*, and *TCF23*) and their proportions in affected DP tumors. (H) Protein-protein interaction network for recurrent, DP-specific deleted or amplified apoptotic genes. Widths and shades of the edges are proportional to the confidence scores of the interactions. Solid lines, interactions within cluster; dashed lines, across clusters. (I and J) (Top) mCherry^+^ human melanoma cells (A375 in I; M407 in J) ± *BAX* knockdown (western blots with TUBULIN as loading control) and their growth patterns in cocultures with HLA-/antigen-specific TCR-transduced primary T cells (E:T ratios, 1:3 in I and 1:5 in J) (top), with control or non-TCR-transduced primary T cells (E:T ratios, 1:3 in I and 1:5 in J) (middle), and without primary T cells but with primary T cell media only (bottom). (K) As in (I), except using mCherry^+^ shBAX-1 A375 cells ± shBAX-1-resistant mutant *BAX* (BAX Mut). (L) As in (I), except using mCherry^+^ M257 cells ± FLAG-DFFA-CR (a dominant-negative mutant) overexpression and an E:T ratio of 1:100. (M) As in (J), except for ± *FAS* knockdown. (N–P) Apoptosis (annexin V and DAPI staining) of indicated cell lines, transduced stably with empty vector, shBAX (N and O), or shFAS (P), after 2 or 3 days of cocultures with primary (control or non-TCR-versus HLA-/antigen-specific TCR-transduced) T cells at E:T ratios in (I), (J), and (M), respectively. Data representative of three (I–M) and two (N–P) independent experiments, with T cells from the same donor. Mean (triplicates) ± SEM (I–P). *p* values, two-way (I–M) or one-way (N–P) ANOVA test. See also Figures S1 and S2 and Tables S1, S2, S3, and S4.

We analyzed WESs (238× average coverage) of patient-matched normal tissues (*n* = 17), baseline tumors (*n* = 17), and AR/DP tumors (*n* = 20) (13 males, 4 females; 8 *BRAF*^MUT^, 7 *NRAS*^MUT^, and 2 *NF1*^MUT^ cutaneous melanomas) (Table S1; Figure S1A). The average tumor mutational burdens of baseline and DP tumors (Table S1) were not different. As CNVs contribute to therapeutic resistance in melanoma,^13,17,43–47^ we detected 14 of 22 copy-number (CN) signatures^48^ in this cohort and CN10/12—focal loss-of-heterozygosity (LOH) signatures recurrent around tumor suppressor genes—only in DP (6 of 20, 30%) but not in baseline tumors (Figure 1A), which suggested single-copy deletions of tumor-suppressing genes as contributors to ICI relapses/recurrences.

We then extracted DP-specific and recurrently (≥three patients) deleted (*n* = 5,035) or amplified (*n* = 2,372) genes (Table S1), both of which were enriched for cell-death regulation (e.g., TP53, TRAF6-mediated IRF7 activation, programmed cell death, and pyroptosis) and IFN signaling, among others (Figures 1B and 1C; Table S2). Next, by collating (from CRISPR-Cas9 screens^20–42^) cancer genes that modulate tumor-cell killing by cytotoxic CD8^+^ T cells or responses to ICIs (Figures S1B–S1E; Table S3), we identified 519 resister (Figure S1B) and 877 sensitizer (Figure S1D) genes (whose knockout promoted resistance and sensitivity, respectively, each with support from ≥2 screens). Both resister and sensitizer genes are enriched for cell-death regulation, IFN signaling, and antigen presentation^7,10,13,15,49^ (Figures S1C and S1E).

We next functionalized clinical DP-specific, recurrent CNV genes by an integrative analysis with preclinical hits from CRISPR-Cas9 screens. 5,053 deleted and 519 resister genes overlapped in 108, whereas 2,372 amplified and 877 sensitizer genes overlapped in 90 (Figures 1D and 1E; Table S3). Moreover, overlapping deleted/resister genes included pro-apoptotic genes (*TNFRSF1A*, *DNASE1L2*, *BAX*, *PPP6C*, *FAS*, *DAPK3*, and *TRAF3*), whereas overlapping amplified/sensitizer genes included anti-apoptotic genes (*BIRC2*, *DAXX*, *MCL1*, *STAT3*, and *TBK1*). Also, both sets of overlapping genes were enriched for apoptotic genes (Figures S1F and S1G; Table S2). Inspection of the 4,927 non-overlapping deleted genes uncovered additional pro-apoptotic genes (*BAD*, *GSDMC*, *DFFB*, *BNIP3L*, *CAPS9*, *APAF1*, and *TP53*). Using a validation clinical cohort, we identified 117 genes that overlapped between DP-specific, recurrently deleted genes and resister genes (Figure S1H) and 277 genes that over-lapped between DP-specific, recurrently amplified genes and sensitizer genes (Figure S1I) (see STAR Methods). Corroborating findings from our cohort, we observed in the validation cohort that the overlapping deleted/resister and amplified/sensitizer genes were enriched, respectively, for pro-apoptotic and anti-apoptotic pathways (Figures S1J and S1K; Table S2).

Due to the limited tumor cohort size, we provisionally nominated significantly mutated genes (SMGs) based on RNA expression in the Cancer Cell Line Encyclopedia. Baseline and DP tumors harbored 111 and 94 SMGs, respectively, with ∼50% overlapping (Figure 1F; Table S4). The baseline-specific SMGs, *FBXO43* and *IPO11*, were sensitizer genes, whereas the DP-specific SMGs, *B2M*, *JAK2*, and *TCF23*, were resister genes. In all DP tumors with predicted loss-of-function, non-synonymous *B2M* and *JAK2* mutations, we observed, respectively, deletion of the other copy or copy-neutral LOH (Figure 1G). Furthermore, one of the two *TCF23-*mutant DP tumors displayed bi-allelic loss-of-function alterations (Figure 1G).

As DP-specific amplifications or deletions overlapped in genomic spans harboring anti- or proapoptotic genes, respectively (Figure S1L), and DP-specific, recurrent CNVs converged on a network of apoptotic genes (Figure 1H), we tested a T cell-protective role of deletions in proapoptotic genes. We set up a coculture assay to measure human melanoma cell line (A375, M407, and M257 engineered to express mCherry-nuclear localization signal or NLS) growth patterns in the presence of primary T cells non-transduced (control) or transduced with an HLA (HLA-A2.1)- and antigen (NY-ESO1)-specific T cell receptor (TCR) (Figure S2A). We then engineered the melanoma cell lines with loss-of-function alterations (*BAX* or *FAS* knockdown or over-expression of a dominant-negative DFFA [DFFA-cleavage-resistant [CR]]^50^) that mimic deletions of pro-apoptotic genes, testing one gene at a time. At the same effector-to-target (E:T) ratio, partial *BAX* knockdown significantly reduced A375 and M407 cytotoxicity by TCR-transduced (but not non-TCR-transduced) T cells from three donors (Figures 1I, 1J, S2B, and S2C). In M407, of the two shRNAs, only the more effective shBAX-2 reduced cytotoxicity by TCR-transduced (but not control) T cells (Figures 1J and S2C). *BAX* knockdown was on-target, as expression of a knockdown-resistant *BAX* rescued resistance to T cells caused by shBAX-1 (and sensitized A375—without shBAX—to T cells) (Figures 1K and S2D). In M257 (but not A375), we observed that DFFA-CR significantly reduced cytotoxicity by T cells (Figures 1L and S2E). In M407, but not A375 or M257, partial *FAS* knockdown attenuated cytotoxicity by TCR-transduced (but not control) T cells (Figures 1M and S2F). Furthermore, we directly measured apoptosis of melanoma cells in cocultures (Figures 1N–1P and S2G–S2J). Expectedly, cocultures with HLA-/antigen-specific (versus control) T cells enhanced melanoma cell apoptosis on days 2 and 3, with early and late apoptosis measured by annexin V + DAPI staining (Figures 1N–1P and S2G). Knockdown of individual proapoptotic genes (*BAX* or *FAS*) partially rescued melanoma cell lines from T cell-induced apoptosis (Figures 1N–1P). Measuring apoptosis by pan-caspase activation, we observed consistent findings (Figures S2H–S2J). Thus, human melanoma cell lines likely display variable apoptotic priming states^51^ that result in distinct thresholds for killing by T cells. This predicts that ICI-unleashed killer T cells would select for gene dosage alterations in distinct sets of apoptotic genes, which converge to enhance apoptotic resistance to killer T cells.

### ICI selects for co-mutations of driver genes

**Figure 2.**
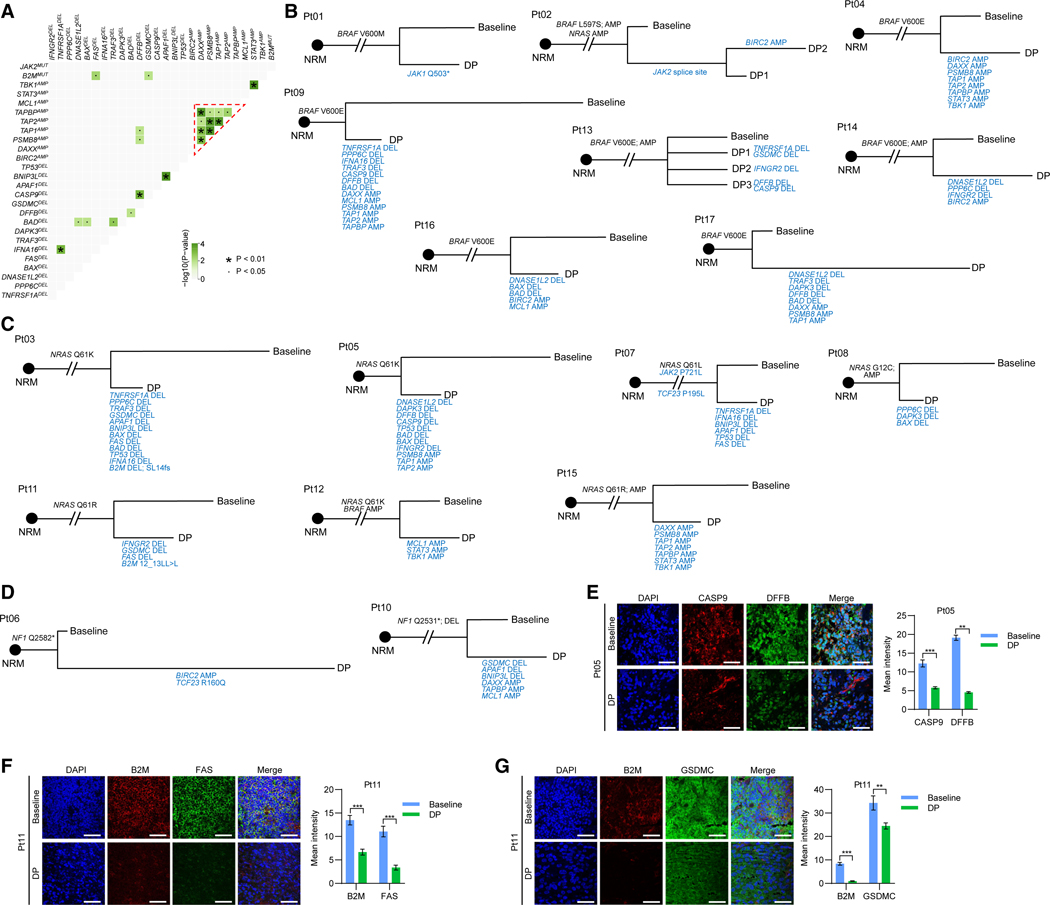
Co-occurring relapse-specific mutations of heterogeneous resistance mechanisms with convergence on apoptotic de-sensitization (A) Correlation analysis of DP-specific CNV and non-synonymously mutated genes. *p* values, pairwise Fisher’s exact test. The red triangle encompasses significant co-occurring pairs of mutated genes within genomic proximity. (B–D) Evolutionary trajectories of *BRAF* (B), *NRAS* (C), or *NF1* (D) mutated melanomas (driver gene mutations in black) of indicated patients. Maximally parsimonious phylogenies based on somatic single-nucleotide variants (SNVs) and insertion or deletions (INDELs) in patient-matched normal (NRM) plus baseline and DP tumors. Resistance-associated CNV and non-synonymously mutated genes in blue. AMP, amplification; DEL, deletion. (E–G) Co-immunofluorescence showing protein levels of indicated resistance-associated genes in patient-matched baseline and DP tumors. Left, representative images (ruler, 50 μm); right, quantifications of eight fields (mean ± SEM; *p* values, unpaired two-tailed Student’s *t* test). See also Figure S3 and Tables S5 and S6.

Consistent with the small and cell context-dependent effect from perturbing a single apoptotic gene (Figures 1I–1P), we observed DP-specific co-deletions (usually single-copy deletions) of pro-apoptotic genes (e.g., *DFFB* and *CASP9* in three DP tumors) and co-amplifications of anti-apoptotic genes (e.g., *BIRC2* and *STAT3* in patient or Pt04, *MCL1* and *STAT3* in Pt12, *TBK1* and *STAT3* in Pt15, and *BIRC2* and *MCL1* in Pt16) (Table S5), which suggested functional cooperativity. Indeed, in a co-occurrence analysis of DP-specific genetic alterations, we identified significant co-occurring gene pairs with loss-of-function (e.g., *B2M* and *GSDMC* or *FAS*, *APAF1* and *BNIP3L*, *BAD* and *TRAF3*, and *DFFB* and *CASP9*) or gain-of-function (*STAT3* and *TBK1*) alterations (Figure 2A; Table S6). To visualize DP-specific co-alterations in each patient, we constructed phylogenies, which uniformly followed branched evolution (Figures 2B–2D). We observed (1) co-deletions in 2–10 pro-apoptotic genes in 12 DP tumors (11 patients), (2) co-amplification of ≥2 anti-apoptotic genes in six DP tumors, and (3) co-occurring amplification of anti-apoptotic and deletion of pro-apoptotic genes in five DP tumors (Figures 2B–2D). Immunofluorescent staining confirmed reduced protein levels in DP tumors with co-deletions of *CASP9* and *DFFB*, *B2M* and *FAS*, as well as *B2M* and *GSDMC* (Figures 2E–2G). We then examined the co-occurrence of DP-specific genetic alterations in three cohorts: (1) largely ICI-naive The Cancer Genome Atlas (TCGA) melanomas (367 tumors or patients), (2) pre-ICI melanomas from our prior^11^ and current studies (56 tumors from 55 patients), and (3) current post-ICI melanomas (20 tumors from 17 patients). Consistently, melanomas that relapsed/recurred on ICI generally displayed significantly higher frequencies of DP-specific mutations and co-mutations (Figure S3).

### AR models recapitulate DP-specific CNVs of apoptotic genes

**Figure 3.**
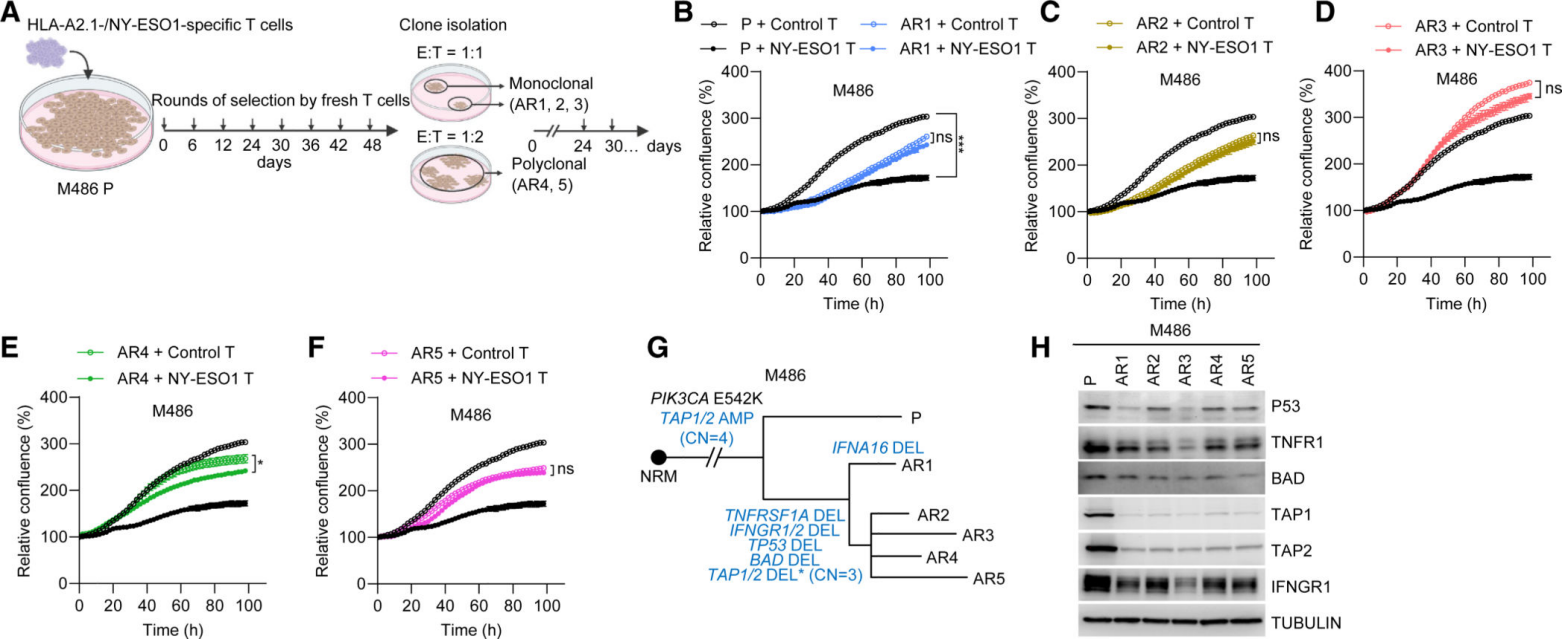
Development of *in vitro* human melanoma AR models that recapitulate clinical DP-specific CNVs of apoptotic genes (A) Schematic of deriving isogenic AR sublines from the M486 P cell line via repeated selection by coculturing with HLA-/antigen-specific T cells. (B–F) Cocultures showing the relative confluence of the M486 P cell line and indicated AR sublines exposed to control (non-transduced) or HLA-/antigen-specific TCR-transduced T cells (E:T ratio of 1:1). Mean ± SEM; *p* values, two-way ANOVA test. Data representative of three replicates with T cells from the same donor. (G) Evolutionary trajectories of the M486 P cell line and its isogenic sublines, AR1–5. Maximally parsimonious phylogeny based on somatic SNVs and INDELs (from bulk WES data) in patient-matched NRM peripheral blood mononuclear cell (PBMC), P, and AR melanoma cells and annotated with CNVs of select genes called using bulk WGS data. Resistance-associated CNV and non-synonymously mutated genes in blue. AMP, amplification; DEL, deletion. (H) Western blots showing the indicated proteins in isogenic M486 P and AR lines. TUBULIN, loading control. See also Figure S4.

Next, we developed *in vitro* AR models based on the coculture system (Figure S2A) using an HLA-A2.1^+^/NY-ESO1^+^ human melanoma cell line (M486) with patient-matched blood to enable somatic genomic analysis. We challenged the parental (P) M486 cells to eight rounds (every 6 days) of HLA-/antigen-specific primary human T cells at a fixed, initial cytotoxic E:T ratio (Figure 3A). After persisters resumed proliferation, we isolated and expanded monoclonal (AR1, AR2, and AR3) and polyclonal (AR4 and AR5) subpopulations. We then validated T cell resistance of M486 AR1–5 sublines (versus the P line) in cocultures by crystal violet staining at endpoints and by real-time detection of melanoma cells engineered with nuclear-localized green fluorescent protein at both E:T ratios tested (Figures 3B–3F and S4A–S4F). By generating and analyzing bulk WGS and WES data, we identified, in 5 of 5 AR sublines, 3,041 AR-specific amplified and 710 deleted genes. Both amplified and deleted genes were enriched for apoptosis or immunogenic cell death (Figures S4G and S4H). M486 AR1–5 (versus M486 P) shared single-copy deletions of pro-apoptotic genes (*TNFRSF1A*, *BAD*, and *TP53*), which recapitulated DP-specific co-deletions in Pt03 (*TNFRSF1A*, *BAD*, and *TP53*), Pt05 (*BAD* and *TP53*), Pt07 (*TNFRSF1A* and *TP53*), and Pt09 (*TNFRSF1A* and *BAD*) (Figure 3G). In addition to a shared *IFNGR1/2* deletion, M486 AR1 harbored a private single-copy *IFNA16* deletion. Western blots confirmed that AR-specific deletions downregulated protein levels (Figure 3H).

**Figure 4.**
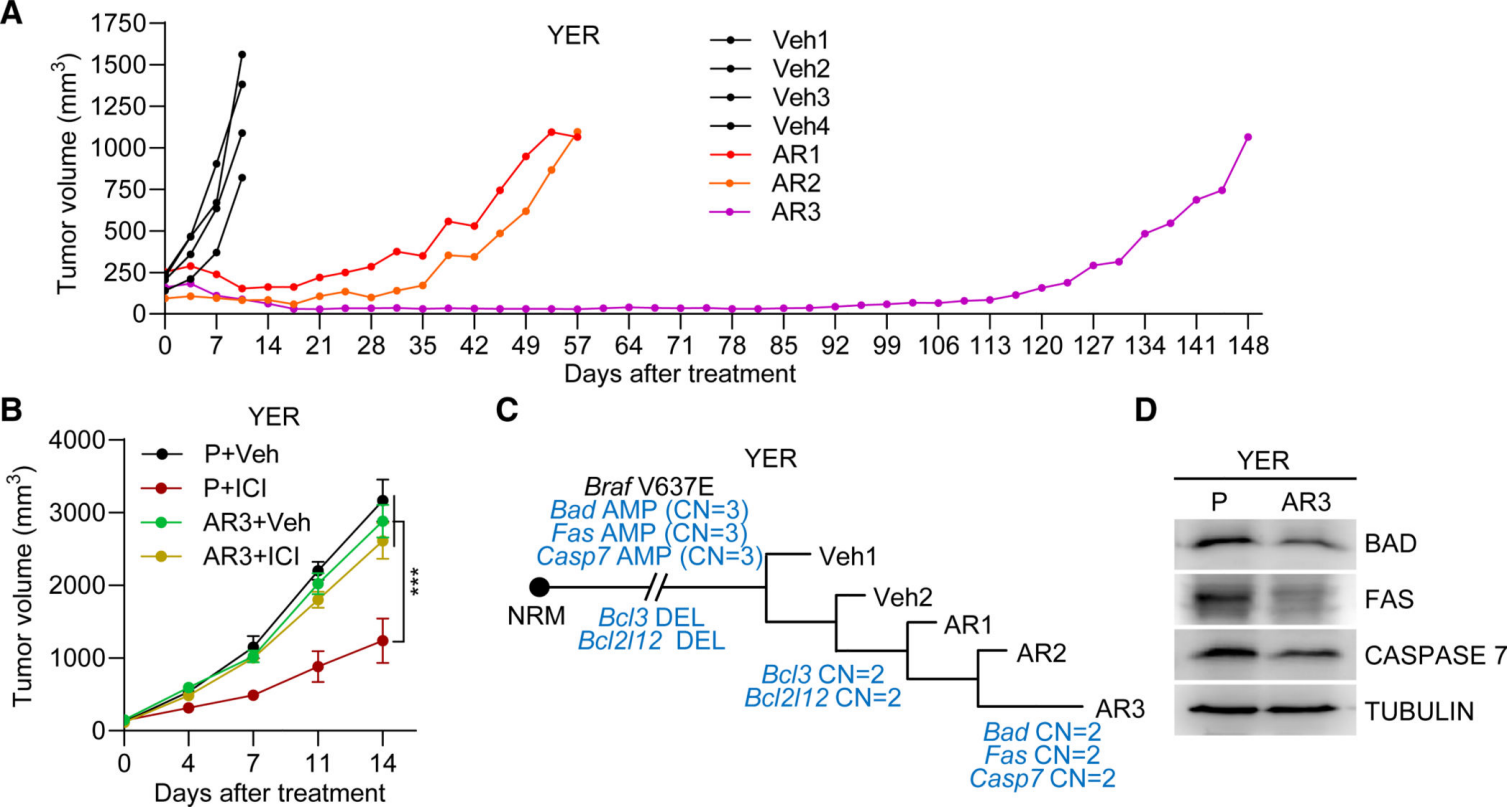
Development of *in vivo* murine melanoma AR models that recapitulate clinical DP-specific CNVs of apoptotic genes (A) Tumor volume curves of individual YER P tumors treated with vehicle (Veh) or ICI (anti-PD-1 + anti-CTLA-4). Three treated tumors that initially shrank and then regrew were selected as AR models. (B) Growth curves of tumors (derived from YER P or AR3-derived cell lines) treated with Veh or ICIs (*n* = 10/group). Data representative of two replicates. Mean ± SEM; *p* values, two-way ANOVA test. (C) As in Figure 3G, except for YER P and AR tumors and phylogenetic tree construction solely by bulk WGS-based CNV calls. (D) Western blots showing the indicated proteins in isogenic YER P and the AR3cl lines. TUBULIN, loading control. See also Figure S4.

We also developed *in vivo* AR models using murine syngeneic *Braf*^V600MUT^ melanoma with high mutational burden (YUMM1.7ER or YER), which responds to anti-PD-1 + anti-CTLA-4 therapy in a CD8^+^ T cell-dependent manner.^52^ We treated YER P tumors with combined ICIs twice a week, selecting for tumors that relapsed after initial tumor regression lasting 25–35 days (AR1, AR2) or >110 days (AR3). From the YER AR3 tumor, we derived a cell line called AR3cl (Figure 4A). We implanted AR3cl into syngeneic hosts and retreated YER AR3cl (versus YER P) tumors, which showed persistent ICI resistance (Figure 4B). By generating and analyzing bulk WGS data, we identified, in ≥2 of 3 AR tumors, 2,743 AR-specific amplified and 178 deleted genes. Both amplified and deleted genes were enriched for apoptosis (Figures S4I and S4J). YER AR1–3 (versus YER P) tumors shared amplification of anti-apoptotic genes (*Bcl3* and *Bcl2l12*), while YER AR3 further displayed deletion of pro-apoptotic genes (*Bad*, *Casp7*, and *Fas*) (Figure 4C). The AR-specific CNVs recapitulated DP-specific co-deletions in Pt03 (*BAD* and *FAS*). Western blots using cell lines and immunofluorescence using tumors confirmed that AR-specific deletions downregulated protein levels (Figures 4D, S4K, and S4L).

### Resistance evolves by subclone-private and preexisting CNVs

**Figure 5.**
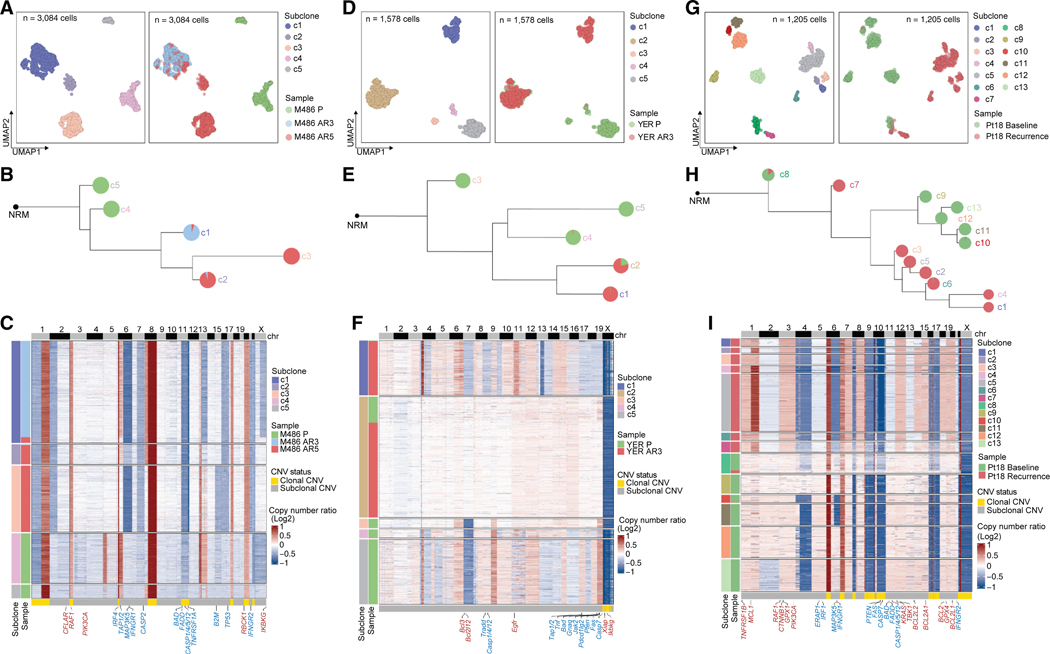
Single-cell WGS uncovers preexisting and *de novo* subclonal resistance evolution (A) UMAPs of single-cell CNV profiles, color-coded by subclones (left) or samples (right, M486 P, AR3, AR5). (B) Evolutionary trajectory with relative branch lengths based on subclonal consensus integer CNV profiles and a diploid normal (NRM) root. Pie charts for each genetic subclone showing the proportions of cells originating from samples in (A). (C) Heatmap based on single-cell CNV profiles of genetic subclones across samples in (A). Genomic regions affected by clonal (gold) and subclonal (gray) CNVs. Genes in red, amplified; genes in blue, deleted. (D–F) As in (A)–(C), except scWGS of YER Veh-treated and ICI-treated AR3 tumors. (G–I) As in (A)–(C), except scWGS of Pt18 baseline and recurrent tumors. See also Figure S5 and Table S7.

To resolve subclonal CNVs ± the selective pressure of tumor-cell killing by T cells, we conducted scWGS (data metrics, Table S7; Figures S5A–S5F) of isogenic cell lines of the human melanoma model (Figures 5A–5C), isogenic murine melanoma tumors (Figures 5D–5F), and patient-matched tumors (Pt #18, Figures 5G–5I). Unbiased clustering of CNV profiles of 3,084 nuclei from isogenic human lines identified 5 subclones (c1–c5), which comprised three M486 AR-specific subclones (c1, c2, and c3) and two M486 P-specific subclones (c4 and c5) (Figure 5A). We then constructed the phylogeny of these subclones by computing the consensus integer CN profiles for each subclone and the lineage relationships using minimum event distance.^53^ The P-specific subclones (c4 and c5) evolved prior to the AR-specific clade (c1–c3). The monoclonal AR subline (AR3) mostly comprised c1, whereas the polyclonal subline (AR5) comprised c1, c2, and c3 (Figure 5B). We used scWGS data to develop an analytic pipeline (see STAR Methods) to cull AR-specific CNVs and CNV-affected genes that were (1) private to a subset of AR-dominant subclones (an AR-dominant subclone defined as comprising >50% of cells from the AR sample[s]) and (2) preexisting in a subset of P/baseline-dominant subclones (defined as comprising >50% of cells from the P or baseline sample) (Table S7). Furthermore, we collated all AR-specific CNV-affected genes that are broadly involved in apoptosis and immune regulation and functionalized them by matching AR-deleted and -amplified genes respectively to resister and sensitizer genes from CRISPR-Cas9 screens (Table S7). We observed that M486 AR-dominant/-specific subclones (c1, c2, and c3) versus P-dominant/-specific subclones (c4 and c5) deleted pro-apoptotic genes (e.g., *IFR4* on chr6p; *MAP3K5* and *IFNGR1* on chr6q; *CASP2* on chr7q; *BAD*, *FADD*, and *CASP1/4/5/12* on chr11q; *TNFRSF1A* on chr12p; *TP53* on chr17p; and *IFNGR2* on chr21q) and pro-antigen-presenting genes (e.g., *TAP1/2* on chr6p and *B2M* on chr15q) (Figure 5C; Table S7). Moreover, AR-specific subclones amplified anti-apoptotic genes (*CFLAR* on chr2q, *RBCK1* on chr20p, and *IKBKG* on chrXq) and genes in characterized innate ICI resistance pathways (e.g., *RAF1* on chr3p and *PIK3CA* on chr3q) (Figure 5C; Table S7).^7,13,54^ Notably, scWGS resolved AR subclone(s)-private gene deletions (e.g., *MAP3K5* and *IFNGR1* in c1 and c3, *TAP1/2* in c2 and c3, and *IRF4* and *B2M* in c3) and amplifications (e.g., *IKBKG* in c2 and c3) (Figure 5C; Table S7).

We also noted that a few cells within the P-dominant subclone, c4, displayed CNs close to the median CNs of some genes (e.g., *BAD* and *B2M*) in the AR-dominant subclones (Figure 5C). Unbiased clustering of CNV profiles of 1,578 nuclei from isogenic mouse tumors identified five subclones (c1–c5), which comprised two YER AR3-dominant subclones (c1 and c2) and three YER P-dominant subclones (c3–c5) or P-specific subclones (c3 and c5) (Figure 5D; Table S7). The P-dominant/-specific subclones (c3–c5) evolved prior to and parallel with the AR3-dominant clade (c1 and c2) (Figure 5E). AR3-dominant (versus P-dominant/-specific) subclones: (1) deleted pro-apoptotic genes (e.g., *Tradd* on chr8; *Casp1/4/12* on chr9; *Tnf* on chr17; and *Bad*, *Jak2*, *Pdcd1lg2* [*Pd-l2*], *Fas*, and *Casp7* on chr19), pro-antigen-presenting genes (e.g., *Tap1/2* on chr17), and *Pten* (a known resister gene on chr19) and (2) amplified anti-apoptotic genes (e.g., *bcl3* and *bcl2l12* on chr7 and *Xiap* and *Ikbkg* on ChrX) and *Egfr* on chr11 (a mutant form of which is linked to innate ICI resistance) (Figure 5F; Table S7). Importantly, AR3-dominant subclones displayed private gene deletions (e.g., *Tradd*, *Casp1/4/12*, and *Tnf* in c1) and amplifications (e.g., *Egfr* and *Ikbkg* in c1 and *Xiap* in c2) (Figure 5F; Table S7). Moreover, AR3-specific amplified or deleted genes were enriched for apoptosis (Figures S5G and S5H). Moreover, AR3-dominant subclonal, clustered gene deletions in chr19 and *Xiap* amplification on ChrX preexisted in the P-dominant subclones c4 and c3, respectively (Table S7).

Unbiased clustering of scWGS-based (Figures S5I and S5J) CNV profiles of 1,205 nuclei from patient (Pt18; Table S1)- matched baseline and recurrent melanomas (the latter of which occurred eight months after and on adjuvant ICI therapy) identified 13 subclones (c1–c13) (Figure 5G), which comprised seven recurrence-dominant subclones (c1–c7, all of which were recurrence-specific except for c6) and six baseline-dominant subclones (c8–c13, all of which were baseline-specific except for c8 and c10) (Figure 5G; Table S7). We found that the post-surgical, recurrent melanoma on adjuvant ICI therapy occurred via both bottleneck and multiclonal evolution, with the recurrence-common ancestor tracing back to the most ancient clone (Figure 5H). Importantly, we observed evidence consistent with the recurrent melanoma displaying apoptotic resistance versus the pre-adjuvant ICI melanoma (Figure 5I; Table S7). For instance, in recurrence-dominant subclones (c1–c7) versus baseline-dominant subclones (c8–c13), we observed chr1q amplification containing the anti-apoptotic gene *MCL1* as well as chr5q, chr11q, and chr21q deletions containing, respectively, the pro-apoptotic genes *IRF1*, *BAD*, and *IFNGR2* (Figure 5I). Incidentally, deletion of chr5q included a gene that promotes antigen presentation, *ERAP1* (Figure 5I), which is also a resister gene (Table S7). Subclone-private apoptotic/ferroptotic resistance likely derived from (1) amplifications in *GPX1*, *TBK1*, *GPX4*, *BCL2L1*, or deletions in *CASP12* (6 of 7 recurrence-dominant subclones); (2) amplifications in *TNFRSF1B* or deletions in *FAS*, *CASP7*, *BCL2A1* (5 of 7 recurrence-dominant subclones); and (3) amplifications in *BCL2L2* and *BCL2* (4 of 7 recurrence-dominant subclones) (Figure 5I; Table S7). Interestingly, recurrence-dominant, subclone(s)-private CNVs resulted in gene amplifications (*RAF1*, *KRAS*) or deletions (*CTNNB1* and *PTEN*) (Figure 5I; Table S7) that have been associated with innate ICI resistance.^7,8,13,54^ Furthermore, a recurrence-specific chr6q deletion harboring the pro-apoptotic (and pro-inflammatory) genes, *IFNGR1* and *MAP3K5*, preexisted in two baseline-dominant subclones, c10 and c11 (Table S7).

### Restoring gene dosage resensitizes AR cells to T cell cocultures

**Figure 6.**
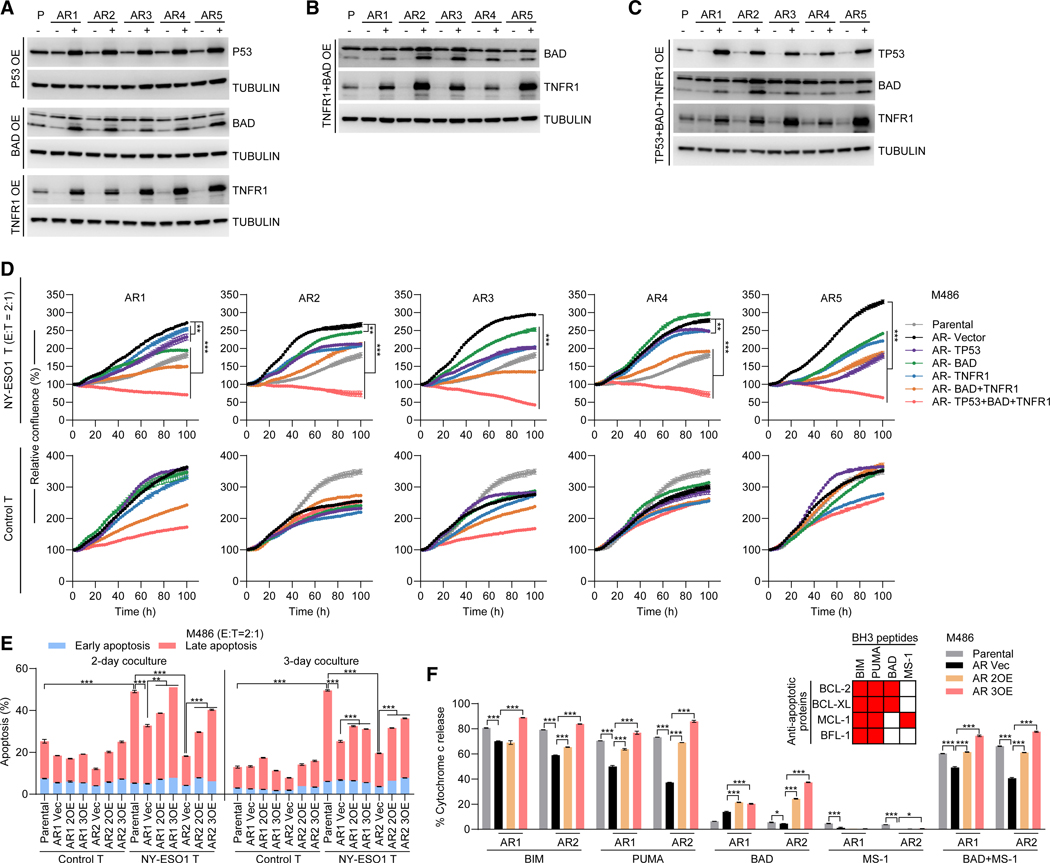
Overexpression of deleted pro-apoptotic genes resensitizes AR melanoma cells to apoptosis induction by cytotoxic T cells (A–C) Western blots of M486 P and AR cells with single (A), double (B), or triple (C) overexpression (OE) of *P53*, *BAD*, and/or *TNFR1*. (D) Cocultures of M486 P and AR cells with indicated overexpressed gene(s) and HLA-/antigen-specific (top) and control (bottom) T cells at an E:T ratio of 2:1. Data representative of three independent experiments with T cells from the same healthy donor. Mean ± SEM; *p* values, two-way ANOVA test. (E) Apoptosis detection (annexin V and DAPI staining) of M486 P, AR1, and AR2 cells transduced with lentivirus containing empty vector (Vec) or two-gene (*BAD* and *TNFR1*) OE (2OE), or three-gene (*P53*, *BAD*, and *TNFR1*) OE (3OE), after 2 or 3 days of cocultures with control (non-TCR-transduced) (left) or HLA-/antigen-specific TCR-transduced (right) primary T cells (left) at an E:T ratio of 2:1. Mean ± SEM; *p* values, one-way ANOVA test. (F) Mitochondrial cytochrome *c* release (measured by flow cytometry) by cells in (E) without T cell cocultures but after triplicate treatments (50 min) with indicated BH3-mimetic peptides (square matrix highlighting the specificity of interacting anti-apoptotic proteins). Peptide concentrations: BIM, 3 μM; PUMA, 30 μM; BAD, 100 μM; MS-1, 10 μM; and BAD + MS-1, 3 μM + 3 μM. Mean ± SEM; *p* values, one-way ANOVA test. See also Figure S6.

To assess the contribution of hemizygous deletions in pro-apoptotic genes (*P53*, *BAD*, and *TNFR1*) to T cell resistance, we engineered in M486 AR1–5 sublines the stable overexpression of (1) *P53*, *BAD*, or *TNFR1* (Figure 6A); (2) *BAD* and *TNFR1* (Figure 6B); and (3) *P53*, *BAD*, and *TNFR1* (Figure 6C). In cocultures (E:T ratio of 2:1 or 1:1), single-gene overexpression in AR sublines generally conferred partial sensitivity (versus the P line) to HLA-/antigen-specific (versus control) T cells, double-gene overexpression conferred partial to full sensitivity, and triple-gene overexpression restored and exceeded the P-line sensitivity (Figures 6D and S6A–S6C). We also measured apoptosis (using flow cytometry to detect annexin V + DAPI or pan-caspase activation) of the M486 AR1 and AR2 sublines, ± double- or triple-gene overexpression, and the M486 P line in similar T cell cocultures (Figures 6E and S6D–S6F). Expectedly, cocultures with HLA-/antigen-specific (versus control) T cells enhanced apoptosis of P cells on days 2 and 3 (Figures 6E and S6D–S6F). Importantly, M486 AR1 and AR2 (versus P) cells displayed a reduced apoptotic response to cytotoxic T cells, which was rescued by the overexpression of deleted pro-apoptotic genes (Figures 6E and S6D–S6F).

### Hemizygous deletions of pro-apoptotic genes reduce apoptotic priming

We also evaluated the melanoma-intrinsic (i.e., T cell-absent) apoptotic priming states by measuring mitochondrial cytochrome *c* release in response to BCL-2 homology 3 domain (BH3)-mimetic peptide treatments of the M486 AR1 and AR2 sublines, ± double- or triple-gene overexpression, versus the isogenic P line (Figures 6F and S6G). The apoptotic priming state is regulated by the net interactions of anti-apoptotic proteins (e. g., BCL-2) with apoptotic activators (e.g., BIM and BID) and sensitizers (e.g., BAD and PUMA). Our BH3-mimetic peptide panel included BIM and PUMA (to measure the overall apoptotic competency and mitochondrial priming state, respectively), BAD (dependency on BCL-2 and BCL-XL), MS-1 (dependency on MCL-1), and BAD plus MS-1 (joint BCL-2, BCL-XL, and MCL-1 dependency). M486 AR1 and AR2 (versus P) cells downregulated mitochondrial cytochrome *c* release in response to BIM or PUMA peptide treatment, which was rescued by the overexpression of deleted pro-apoptotic genes (Figure 6F). In both P and AR cells, cytochrome *c* release in response to individual BAD (100 μM) or MS-1 (10 μM) peptide treatment was weak, but treatment with a combination (BAD, 3 μM; MS-1, 3 μM) of peptides released the apoptotic block (Figure 6F). In this context, both AR sublines (versus the P line) displayed reduced apoptotic competency, which was restored by overexpression of hemizygously deleted pro-apoptotic genes (Figure 6F).

### Restoring apoptotic priming resensitizes AR tumors to ICI therapy

**Figure 7.**
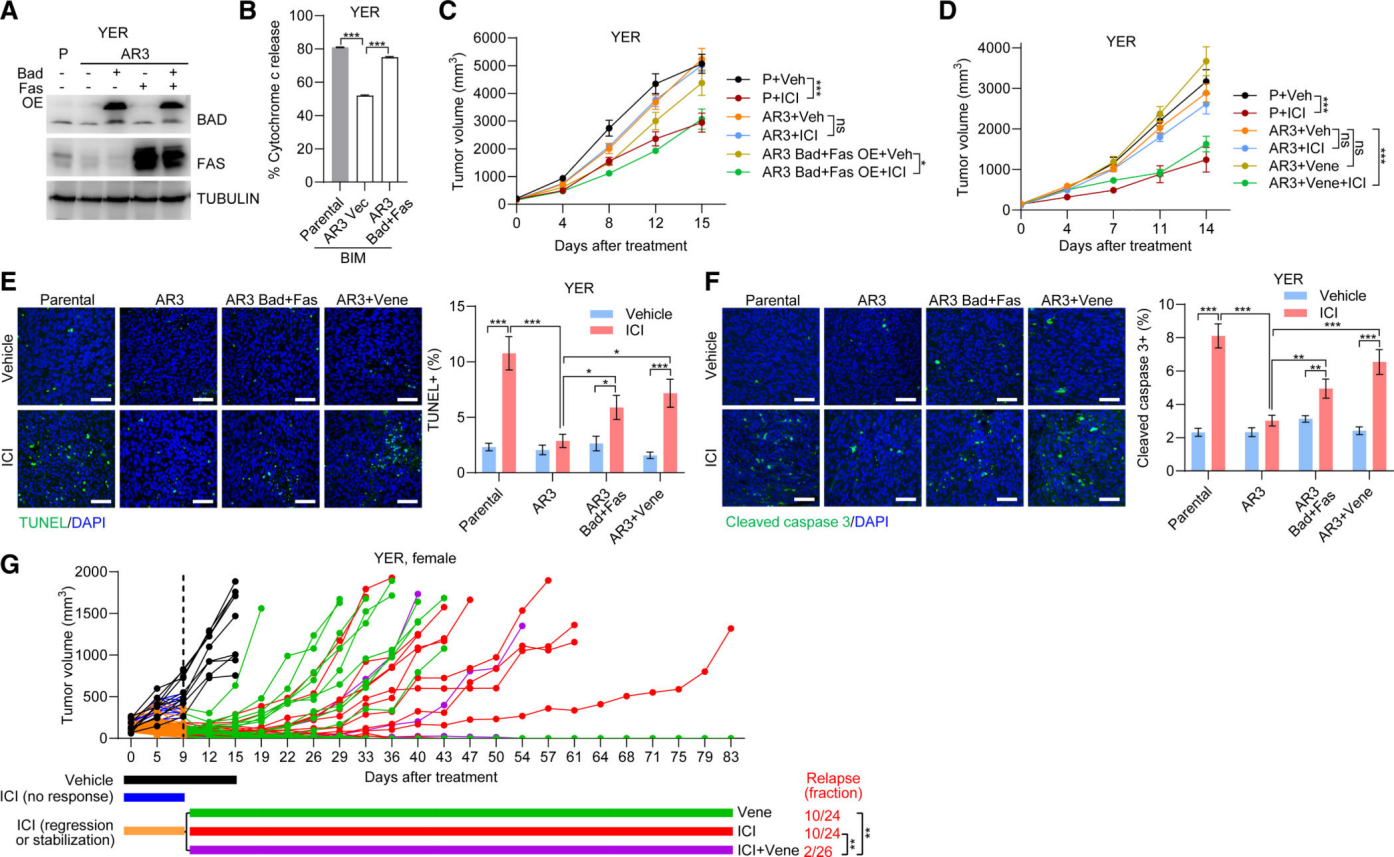
Enhancing apoptotic priming resensitizes AR melanoma tumors to ICIs and prevents relapses in ICI persisters (A and B) Western blots (A) and BH3 profiling (BIM 10 μM, 75 min, in duplicates) (B) of the YER P cell line and AR3cl ± *Bad* and *Fas* OE (Vec, empty vector). Mean ± SEM; *p* values, one-way ANOVA test in (B). (C and D) Tumor volume curves in response to vehicle (Veh) or ICI (anti-PD-1 + anti-CTLA-4) therapy of YER P-derived tumors or AR3cl-derived tumors (±*Bad* and *Fas* OE in C or ± venetoclax [Vene] treatments in D) (*n* = 5 mice or 8–10 tumors per group). Mean ± SEM; *p* values, two-way ANOVA test. (E and F) TUNEL (E) and cleaved caspase-3 (F) staining of tissues (collected on day 5) from indicated YER tumors in (C) and (D). Representative images (left; ruler, 50 μm) and quantifications (right) of 10 high-power fields. Mean ± SEM; *p* values, one-way ANOVA test. (G) Growth curves of individual YER tumors (in sex-mismatched or female Jackson Laboratory C57BL/6J mice, *n* = 110 tumors) treated with vehicle or ICIs (anti-PD-1 + anti-CTLA-4). On day 9 after initiating ICI treatments, responding (defined as tumor volume stabilization or regression) tumors on both flanks of mice were assigned to three indicated groups. Relapse rates (relapse tumor defined as two consecutive increases in tumor volume) are shown. *p* values, Fisher’s exact test. See also Figure S7.

Next, we turned to the YER AR tumor model to interrogate deletion-driven loss of apoptotic priming as a mechanism of resistance evolution. We first engineered YER AR3cl to overexpress stably *Bad* and *Fas* (Figure 7A). Using the human BIM BH3-mimetic peptide that is active in mouse cells, we found that YER AR3cl (versus P) cells downregulated mitochondrial cytochrome *c* release upon BIM peptide treatment, and overexpression of *Bad* + *Fas* rescued the apoptotic defect (Figure 7B). *In vivo*, *Bad* + *Fas* overexpression resensitized YER AR3 tumors to ICI therapy (Figures 7C and S7A), which was phenocopied by treatment of YER AR3 tumors (without overexpression) with both ICIs and venetoclax, a BCL-2-selective BH3-mimetic (i.e., a BCL-2 inhibitor) (Figures 7D and S7B–S7D). By terminal deoxynucleotidyl transferase-mediated dUTP nick-end labeling (TUNEL) (Figure 7E) or cleaved caspase-3 (Figure 7F) immunofluorescent staining, we consistently observed that, while ICI therapy induced apoptosis in YER P tumors, it failed to do so in YER AR3 tumors. However, either *Bad* + *Fas* overexpression or venetoclax cotreatments resensitized AR3 tumors to apoptosis induction by ICIs (Figures 7E and 7F).

### Augmenting apoptotic priming in ICI persisters prevents tumor relapses

Finally, we evaluated whether venetoclax cotreatment of ICI-induced persisters can mitigate the selective pressure to escape from ICI. We designed *in vivo* studies such that venetoclax cotreatments begin *after* ICIs have achieved tumor growth stabilization or regression. First, we tested whether implanting male YER P cells into sex-mismatched female syngeneic mice would enhance the ICI response rate. We observed that ICIs in female (versus male) mice elicited a higher response rate (Figures S7E and S7F), and ∼30% of YER P tumors in female mice relapsed after prolonged ICI treatments (Figure S7F). Second, in independent experiments (*n* = 110 tumors in Figure 7G; *n* = 52 tumors in Figure S7G), we selected 75–250 mm^3^ tumors for vehicle versus ICI therapy (day 0) and, on days 9 or 10, assigned only tumors that stabilized or shrunk after ICI therapy into three groups: (1) ICI off, venetoclax on; (2) ICI on, venetoclax off; and (3) ICI on, venetoclax on. Importantly, ICI + venetoclax-cotreated ICI persisters relapsed at the lowest rate, versus the venetoclax-only or ICI-only group (Figures 7G and S7G). In fact, venetoclax-only (versus ICI-only) treatment resulted in a higher relapse rate (*p* = 0.033, Fisher’s exact test). ICI + venetoclax co-treatment (versus ICI alone) did not negatively impact the weight gains of mice (Figures S7H and S7I).

## DISCUSSION

We integrated analysis of somatic mutations specific to relapsing (advanced disease setting) or recurrent (resectable, adjuvant setting) clinical melanomas after ICI therapy with analysis of cancer genes that regulate cytotoxicity by T cells. This comparative analysis nominated patient-recurrent and DP-specific CNV genes that drive acquired ICI resistance and identified CNV convergence on apoptotic genes. Specifically, relapsing or recurrent melanomas frequently feature co-occurring CNVs with distinct permutations of (usually) hemizygous deletions (of pro-apoptotic genes) and/or amplifications (of anti-apoptotic genes). Hemizygous gene deletions or LOHs at multiple loci are consistent with a model of cumulative haploinsufficiencies of tumor suppressor genes.^55–57^ Thus, ICIs select for multiple genetic hits in distinct apoptotic (and perhaps pyroptotic/ferroptotic) genes, which results in resistance to T cell attack^58^ and/or avoidance of T cell recognition.^59^ Tumor-cell killing by cytotoxic T cells occurs as death by many cuts or additive sublethal attacks.^60^ Thus, DP on ICIs may necessitate raising the apoptotic threshold to varying extents, depending on distinct intratumoral immune microenvironments (e.g., quantitative and qualitative properties of cytotoxic T cells) and pretreatment, tumor-cell-intrinsic, apoptotic priming states. Our single-cell CNV analysis highlighted the multi-factorial and subclonal mechanisms underlying heterogeneous DP states.

Although clinical relapses/recurrences after ICI therapy follow highly variable kinetics and metastatic patterns, the consensual definition of AR to ICI refers to DPs in patients who have had a period of initial response to ICI therapy. From here, variations of this definition center around the depths (stable disease, partial or complete response) of the initial response, durations of the response (where a delayed relapse is defined as ≥6 months of response to differentiate AR from primary resistance), and ICI treatment continuation/discontinuation status. DP in the current cohort of eighteen patients mostly conformed to the general criteria of partial/complete responses, delayed relapses, and DP while on treatment. However, three patients (Pt#16–18) were treated with ICI therapy in the post-surgical or adjuvant setting, where an initial ICI response was unevaluable. In one of these three patients, adjuvant ICI treatment was stopped after 2 months of initiation due to toxicities, and her disease recurred after 3 years of initiating adjuvant ICI treatment, which was long after ICI discontinuation. The recurrent melanomas from all three adjuvant ICI-treated patients harbored CNVs affecting multiple apoptotic genes that were absent in their resected, patient-matched, ICI-naive melanomas. These observations suggest a CNV-driven AR mechanism similar to that observed in advanced, non-resectable melanomas. Thus, identifying relapse/recurrence-specific genomic alterations and their affected genes/pathways may be more impactful in advancing our knowledge of resistance evolution than adhering to any specific definition. In support of this view, CN loss burden associates with innate ICI resistance or lack of clinical benefit,^61^ suggesting a continuum of resistance mechanisms from the innate to acquired settings.

Tumor-cell killing by T cells requires the pro-apoptotic effects of perforin and granzymes, death receptors, and/or IFNs.^59,60^ To evolve resistance to killing by ICI-invigorated T cells, melanoma CNVs downregulated both extrinsic and intrinsic programmed cell-death mechanisms, attenuating IFN-γ–tumor necrosis factor alpha (TNF-α)–FAS immunogenic effector signaling and tumor-cell-intrinsic BAD-BAX-APAF1-CASP9 signaling. Moreover, *DFFB* deletion in tumor cells may attenuate killing by granzymes, as inactive DFFB is cleaved and activated by granzymes B and M. *BAD*/*BAX* deletions in tumor cells may also blunt rapid killing by granzyme B, which activates BAX and caspase-3. The terminal executioner of apoptosis, caspase-3, can cleave and activate pore-forming gasdermin proteins, leading to an inflammatory form of programmed cell death (pyroptosis).^62^ *GSDMC* deletion may weaken the link between apoptosis and pyroptosis. Translationally, our results suggest that blocking anti-apoptotic proteins (e.g., BCL-2 and BIRC2) or activating pro-apoptotic proteins (e. g., BAX) may reverse or even prevent acquired ICI resistance.

Most CRISPR-Cas9 screens utilize cancer cell lines and focus on CD8^+^ T cells, thereby providing no insights into clinical ICI relapse caused by defects in responsiveness to other cytolytic immune cell types or immune-cell trafficking. Also, such CRISPR-Cas9 screens do not explain the evolutionary origins or genomic-instability mechanisms of resistance. Our prior studies^11,13,17,45–47^ highlighted the importance of CNV as a genomic-instability mechanism in melanoma therapeutic resistance, and this study points specifically to the critical role of CNVs in the evolution of ICI resistance. CNVs may be less immunogenic than non-synonymous small-scale mutations, thereby facilitating an evolutionary pathway of lesser resistance. Going forward, this study challenges us to identify the mutational forces underlying CNVs and additional convergent phenotypes that may be pharmacologically actionable.

### Limitations of the study

We prioritized tumor tissues for genomic analysis. Therefore, other informative profiles such as RNA sequencing (RNA-seq) are unavailable. Also, SMG analysis was limited by the current cohort size. Since we relied on bulk WES over bulk WGS in this study, we could not identify the origins of relapse-driving CNVs, larger scale and complex genomic-instability mechanisms, and their mutational forces. We also could not carry out allele-specific copy-number analysis. Thus, we may have missed resistance genes potentially co-altered by somatic SNVs/IDs and CNVs. The coculture model used only T cells, which precluded analysis of escape mechanisms from other tumor-cytolytic cell types. The current clinical cohort has limited representation of rare melanoma subtypes and the spectrum of metastatic organ sites. Development of additional AR models should enable identification of distinct resistance mechanisms and the potential mechanistic basis of their co-occurrence. The model of CNV-driven apoptotic and ICI resistance may not be generalizable to ICI relapses in other cancer histologies.

## STAR★METHODS

### RESOURCE AVAILABILITY

#### Lead contact

Further information and requests for resources and reagents should be directed to the lead contact, Roger S. Lo (rlo@mednet.ucla.edu).

#### Materials availability

Requests for cell lines and plasmids should be directed to the lead contact.

#### Data and code availability

- The aligned BAM files of bulk sequencing data as well as raw scWGS data have been deposited in public databases with accession numbers listed in the key resources table.
- This paper does not report original codes.
- Additional information required to reanalyze the data reported in this paper and MAF files is available from the lead contact upon request.

### EXPERIMENTAL MODEL AND PARTICIPANT DETAILS

#### Human samples

All patients with metastatic cutaneous melanoma were treated at the University of California, Los Angeles (UCLA), Vanderbilt-Ingram Cancer Center, and California Pacific Medical Center. All (except three patients on adjuvant ICI therapy) had an objective (partial or complete) response to anti-PD-1 or anti-PD-1 + anti-CTLA-4 blocking antibodies, based on Response Evaluation Criteria in Solid Tumors^63^ and immune-related response criteria.^64^ After ≥ six months of objective responses (except in Pt16–18 treated with adjuvant ICI), in situ or de novo relapsing tumors (on or off active therapy) were considered disease progression with acquired resistance. Patient-matched longitudinal tumor biopsies (baseline, *n* = 18; disease progressive or recurrent tumors, *n* = 21; patients, *n* = 18) were from the same or distinct anatomic sites. We used frozen whole blood, peripheral blood mononuclear cells (PBMCs), or tumor-uninvolved tissues (e.g., lymph nodes) as sources of patient-matched normal tissues. Whole-exome sequence (WES) data from patients one to four were generated previously.^15^ Tissue collection and genomic analysis were approved by local institutional review boards with informed consent from each patient.

#### Mice

We used male or female C57BL/6ROC mice (UCLA Radiation Oncology breeding colony) or C57BL/6J (The Jackson Laboratory) at 6–8 weeks of age. All animal experiments were conducted according to the guidelines approved by the UCLA Animal Research Committee.

#### Cell lines

All human and murine cell lines were negative for mycoplasma (Lonza) and identified by RNA-seq and/or GenePrint 10 system (Promega) at periodic intervals. All human cell lines were cultured in either RPMI (A375, M407, and M257) or DMEM (M486, HEK293T) supplemented with penicillin/streptomycin, 2 mM glutamine, 10% heat-inactivated FBS (Omega Scientific or Gibco) in a humidified 37° C incubator with 5% CO_2_. The murine cell line, YUMM1.7ER (YER), and the derived subline YER AR3cl were cultured in DMEM/F12 supplemented with non-essential amino acids (Gibco), penicillin/streptomycin, amphotericin B (Gibco), and 10% heat-inactivated FBS (Omega Scientific or Gibco) in a humidified 37° C incubator with 5% CO_2_.

### METHOD DETAILS

#### Bulk WES analysis of clinical tumors

We extracted gDNAs from formalin-fixed paraffin-embedded (FFPE) tumors or tumor-uninvolved tissues using the QIAGEN QIAamp DNA FFPE Tissue Kit; snap-frozen tumors using the QIAGEN AllPrep DNA/RNA Mini Kit; and patient-matched PBMCs, whole blood, or human melanoma cell lines using the QIAGEN FlexiGene DNA Kit. We prepared whole-exome libraries using the Roche NimbleGen Exon-Seq Kit, the Roche NimbleGen Seqcap Kit, or the Roche KAPA HyperPlus Library Preparation Kit with the KAPA HyperCap Workflow v3.0 for exome hybridization. We paired-end sequenced pooled libraries with a read length of 2 × 150 bp on the Illumina HiSeq 3000, Illumina NovaSeq 6000 S4, or X Plus platforms.

For somatic mutation calling, we used BWA-MEM for mapping, SAMtools for sorting, and Picard for the removal of duplications. We identified somatic single-nucleotide variants (SNVs) and small insertion-deletions (INDELs) of tumors as we previously reported.^13^ Specifically, we called SNVs using a combination of the Unified Genotyper tool of GATK, MuTect, and VarScan2 and called INDELs using a combination of GATK-UGF, SomaticIndelDetector of GATK (IndelLocator), and VarScan2. For each detection tool, SNVs/INDELs were supported by at least five reads in the tumors and none in the patient-matched normal tissues. We then used Oncotator to annotate somatic SNVs/INDELs. We used Sequenza to detect tumor purities and ploidies with default parameters. We called CNVs using the union of CN calls derived from Sequenza and VarScan2.

We generated the CNV profiles of baseline and DP tumors from each patient using SigProfilerAssignment with default parameters to decipher CN signature compositions, with COSMIC CN signatures version 3.3 as reference. We evaluated the associations of gene deletions or co-mutations with the CN10 signature using the two-sided Fisher’s exact test of CN10 non-attributed or attributed (= 0, > 0) against gene deletions or co-mutations presence or absence. For the current clinical cohort, we defined recurrent DP-specific gene amplifications or deletions as ≥ three patients.

We identified DP-specific SMGs using MutSig2CV with each patient’s mutational profile of a single DP tumor or a combination of multiple DP tumors (if applicable). For a patient with multiple DP tumors, we considered a mutation to exist in this patient if it was detected in ≥ one tumor. Each baseline tumor’s mutational profile was used as input to identify baseline-specific SMGs. To circumvent the limitation of a small cohort, we inflated type I error by not performing multiple testing and identified MutSig2CV genes at *P* values < 0.05. To reduce false-positive SMGs, we nominated provisional SMGs by requiring positive RNA expression (mean log_2_CPM > 0) of given genes in the Cancer Cell Line Encyclopedia RNA expression database.

We analyzed the co-occurrence of mutated genes using the *somaticInteractions* function in the R package ‘maftools,’ which performs pair-wise Fisher’s exact test to identify significantly co-altered or mutually exclusive gene pairs. To account for multiple hypothesis testing, the function applies the Benjamin-Hochberg procedure to control the false discovery rate. Although data visualization highlights gene pairs with *P* values < 0.05 and odds ratios > 1, the underlying analysis includes multiple hypothesis testing. We also calculated the patient-level frequencies of co-mutations in distinct tumor cohorts. For the TCGA-SKCM ICI-naïve cohort, we downloaded the CNV data from cBioPortal. The WES data of a pre-ICI or ICI-naive melanoma cohort have been reported.^11^ When multiple tumors were available from a given patient, they were considered as an entirety, e.g., a deleted gene was counted in a given patient if it were identified from one of the patient-matched tumors.

For phylogenetic analysis, we used the PHYLIP program with the parsimony algorithm, as we previously reported.^13,43,45,46^ We annotated each tree with truncal driver mutations and putative driver genetic alterations of ICI resistance.

#### Bulk WES and/or WGS analyses of experimental models

We extracted gDNAs from human melanoma cells and mouse tumors using the QIAGEN AllPrep DNA/RNA Mini Kit. We constructed whole-genome libraries using the Roche KAPA HyperPrep Kit. From the pooled indexed libraries, we generated paired-end sequences with a read length of 2 × 150 bp using the Illumina NovaSeq X Plus platform. Whole-exome libraries were constructed from whole-genome libraries using the KAPA HyperCap Workflow v3.0 for exome hybridization. From the pooled indexed libraries, we generated pair-end sequences with a read length of 2 × 150 bp using the Illumina HiSeq 3000, NovaSeq S4, or X Plus platforms.

We aligned the paired-end WES reads from the isogenic human melanoma M486 P line and AR sublines to the human reference genome (hg19) using BWA-MEM. The resulting alignments were processed with SAMtools and Picard to sort the alignments and remove PCR duplicates. We called SNVs using a combination of GATK’s Unified Genotyper, MuTect, and VarScan2. We called INDELs through a combined analysis using GATK-UGF, SomaticIndelDetector (IndelLocator) from GATK, and VarScan2. We required SNVs and INDELs to be called by > five reads in the cell line samples and 0 reads in the patient-matched PBMC. We annotated somatic SNVs and INDELs using Oncotator and constructed the phylogeny using the PHYLIP program. Moreover, we aligned the paired-end WGS reads from the M486 isogenic lines to the human reference genome (hg19) using BWA-MEM. We called CNVs using CNVkit with default settings. We then annotated the M486 phylogenetic tree with CNVs of select genes and verified the absence of CNVs of these genes using WGS derived from the M486 patient-matched PBMC.

For the syngeneic murine melanoma AR model (YER), we aligned paired-end WGS reads to the mouse reference genome (mm10) using BWA-MEM. The resulting alignments were processed with SAMtools and Picard tools to sort the alignments and remove PCR duplicates. Due to the absence of a true YER tumor-matched normal tissue, we called CNVs using CNVkit. Using these CNV profiles as input, we ran MEDICC2^53^ to construct a phylogenetic tree based on minimum event distances.

#### STRING analysis

We used the STRING database (version 12.0) to visualize potential interactions among proteins encoded by recurrent, DP-specific, amplified or deleted apoptotic genes. A median confidence score of 0.4 was used as the threshold. We excluded disconnected nodes. The k-means algorithm defined functional clusters.

#### Cataloging resister and sensitizer genes from CRISPR-Cas9 screens

We combined data from 23 published studies of functional CRISPR-Cas9 screens (Table S3) to identify genes that modulate tumor cell killing by T cells (excluding CAR T cells) or ICI treatments, in vitro and in vivo, in human or mouse models. We identified these studies by a PubMed search using the keywords: CRISPR screening, tumor-intrinsic, T cell killing, immune evasion, and immunotherapy target. We categorized screen hits into resister genes (whose loss-of-function/knockout promoted resistance or gain-of-function/CRISPR activation promoted sensitivity) and sensitizer genes (whose loss-of-function/knockout promoted sensitivity or gain-of-function/CRISPR activation promoted resistance), according to the specific cutoffs defined by each study. We defined sensitizer or resister genes as those hits with concordant evidence in ≥ two independent measurements.

#### Immunofluorescence

FFPE tissue sections were heated at 60° C for 30 minutes and then immersed in xylene, followed by ethanol-gradient solutions for deparaffinization and rehydration. For antigen retrieval, we heated tissue sections in Tris-EDTA buffer (pH 9.0) (Abcam) at 95° C for 15 minutes. We permeabilized tissues in 0.4% Triton X-100 in PBS for 15 minutes, followed by blocking with 10% normal goat serum in PBS for 1 hour. Tissues were incubated with validated primary antibodies, including anti-B2M (Invitrogen, 1:200), anti-GSDMC (Invitrogen, 1:100), anti-FAS (Invitrogen, 1:100), anti-DFFB (Invitrogen, 1:100), anti-CASP9 (Invitrogen, 1:100), and anti-Bad (CST, 1:100), and anti-cleaved CASP3 (CST, 1:500) at 4° C overnight. We then incubated tissues with goat anti-mouse IgG (highly cross-absorbed secondary antibody, Alexa Fluor Plus 488, Invitrogen, 1:400) or goat anti-rabbit IgG (highly cross-absorbed secondary antibody, Alexa Fluor 555, Invitrogen, 1:400). Nuclei were counterstained with DAPI (Sigma-Aldrich). For TUNEL staining, after the afore-mentioned deparaffinization and rehydration steps, tissues were incubated with Proteinase K (CST, 20 μg/ml) solution for 30 min at 37° C and then rinsed two times in PBS for 5 min each. Tissues were then incubated with 100 μL TUNEL Equilibration Buffer (CST) for 5 min, followed by 50 μL of TUNEL reaction mix (1 μL of TdT Enzyme in 50 μL of TUNEL Reaction Buffer) for 4 hours at 37° C. We then rinsed the samples three times in PBS-TB for 5 min each and counterstained the nuclei with DAPI. We imaged stained tissues with a Leica confocal SP8 Light-Sheet microscope and quantified fluorescence intensity by ImageJ (version, 1.54d).

#### Peripheral T cell transduction

For peripheral T cell transduction, we used the NY-ESO1 TCR retroviral vector (Antoni Ribas, UCLA) and generated retrovirus by co-transfection of HEK-293T with pMSGV, pRD114, and pHIT using BioT (Bioland Scientific). Fourteen hours after transfection, we treated cells with 10 mM sodium butyrate (Thermo Fisher) and 20 mM HEPES (Gibco) in fresh media for 6–8 hours and harvested retroviral supernatants 48 and 72 hours after transfection. We isolated human primary T cells from PBMCs of healthy donors (UCLA Virology Core) by negative selection using the Pan-T Cell Isolation Kit (Miltenyi Biotec) and magnetic LS columns (Miltenyi Biotec). We cultured isolated T cells in RPMI 1640 supplemented with 10% fetal bovine serum (Omega Scientific or Gibco) and 50 IU/ml human IL2 (Miltenyi Biotec) and activated the primary T cells on the same day with 25 μL CD3/CD28 dynabeads (Gibco) per million T cells. Two days after activation, cells were transduced with retrovirus harboring NY-ESO1 TCR in 6-well plates pre-coated with RetroNectin (Takara). Media was replenished every 2–3 days. Five days after transduction, we measured the proportion of NY-ESO1 TCR-positive T cells by flow cytometry using anti-human CD3 (BioLegend) and anti-human TCR Vβ13.1 (BioLegend) antibodies. CD3/CD28 dynabeads were removed using magnetic isolation 7 days after activation. Engineered T cells were freshly used or cryopreserved using Cryostor CS10 medium (Biolife Solutions) 12–14 days after activation.

#### Tumor cell-T cell coculture

For tumor cell-T cell cocultures, mCherry-NLS- or H2B-GFP-labeled melanoma cells were seeded at a concentration of 4 × 10^4^ per well in 96-well plates and incubated overnight. NY-ESO1 TCR-transduced T cells were added the following day at the indicated E:T ratios with triplicates per group. Plates were imaged using the IncuCyte SX5 real-time, live-cell imaging system (Sartorius) with the 10x objective at 2-hour intervals for 4 days. Cocultures with non-transduced T cells or cultures with only T-cell media served as negative controls. We analyzed the integrated mCherry or GFP count per well as quantitative measurements of live melanoma cells. All measurements were normalized to the start-time signals.

#### Transduction of human and murine melanoma cell lines

We subcloned mCherry-NLS (Addgene) into the pLV-neo lentiviral vector, H2B-GFP (Addgene) into a pLV-blast vector, and generated lentiviruses. We then transduced human melanoma cell lines to express mCherry-NLS or H2B-GFP after neomycin (Sigma) or blasticidin (Gibco) selection, respectively, for one week. The FLAG-tagged caspase 3 cleavage-resistant DFFA (DFFA-CR)^50^ in a lentiviral vector (pHAGE_EF1a) was obtained from Stephen Elledge via a material transfer agreement (Dana-Farber Cancer Center). The cDNAs of human *BAX*, *BAD*, *P53*, and *TNFR1* were obtained from the UCLA Molecular Screening Shared Resource and subcloned into pLV-blast, pLV-hygro, pLV-puro, and pLV-neo vectors, respectively. Mouse *Bad* and *Fas* genes were PCR amplified from the cDNA of YUMM1.7ER cells and inserted into pLV-neo and pLV-blast vectors, respectively. shRNA sequences for human *FAS* and *BAX* (UCLA Molecular Screening Shared Resource) are: ACAAACTTCATCAAGAGTA (shFAS-1), GGCTTAGAAGTGGAAATAA (shFAS-2), CGGAACTGATCAGAACCATCA (shBAX-1), and GCCAGCAAACTGGTGCTCA (shBAX-2). We generated vector control, shRNA, or cDNA lentiviruses by cotransfection of the above constructs with pMD2.G, pRSV-Rev and pMDLg/pRRE into HEK 293T cells using calcium phosphate. We transduced human and mouse cell lines with lentiviruses in the presence of 10 μg/mL polybrene (Millipore) for 48 hours and performed antibiotic selection for one week. We introduced synonymous mutations (A393G, C394T, G396A and C399T) into the shBAX target sequence within the human *BAX* cDNA using PCR mutagenesis and subcloned this mutant human *BAX* cDNA into the pLV-blast lentiviral vector. We then transduced the *BAX* knocked-down cell line with the knockdown-resistant, mutant *BAX* virus to rescue *BAX* expression.

#### Apoptosis detection

In the annexin V/DAPI apoptosis assay (Invitrogen), we trypsinized cells and washed them with cold PBS two times. Cells were then resuspended in 100 μL annexin V binding buffer and stained with annexin V conjugate with 1 μg/ml DAPI for 15 minutes. Stained cells were analyzed by flow cytometry (Attune NxT, ThermoFisher). We detected early (annexin V^+^/DAPI^-^) and late (annexin V^+^/DAPI^+^) apoptosis in mCherry^+^ or GFP^+^ tumor cell populations. In the assay using the Poly Caspase Staining Kit (Abcam), cells were trypsinized; washed with cold PBS two times; resuspended in 295 μL PBS containing 10% FBS; and stained with 5 μL 660-VAD-FMK working solution for 60 minutes at 37°C. We then washed cells with Apoptosis Wash Buffer two times, resuspended cells in 500 μL Apoptosis Wash Buffer, and analyzed activated pan-caspase levels in mCherry^+^ or EGFP^+^ tumor cell populations by flow cytometry (Attune NxT, ThermoFisher).

#### Murine tumor studies

We injected subcutaneously one million YUMM1.7ER cells, AR3cl cells (derived from the YER AR3 tumor), or AR3cl cells engineered with *Bad*+*Fas* overexpression on both flanks of syngeneic mice. We measured tumors with a caliper every 2–3 days to calculate the tumor volumes based on the formula (length × width^2^)/2. Once tumors reached volumes of 100–250 mm^3^, we assigned mice randomly into experimental groups. We treated tumor-bearing mice by injecting anti-PD-1 (Leinco) and anti-CTLA-4 (Bio X Cell) intra-peritoneally twice a week (300 μg/mouse for each antibody for the first two weeks and then 200 μg/mouse for the remainder time). Venetoclax was formulated in 10% DMSO + 40% PEG 300 + 5% Tween 80 + 45% saline and administered at 25 mg/kg/day via gavage.

#### Generation of acquired-resistant cell lines or tumors

To generate human melanoma sublines with acquired resistance to T cells, we seeded M486 cells at 2.5 × 10^5^ per well in 12-well plates and, after overnight cultures, treated M486 cells continuously with fresh NY-ESO1 TCR-transduced T cells every 6 days for 8 rounds until the formation of single cell-derived colonies. Monoclonal sublines (AR1, AR2, AR3) were derived using an E:T ratio of 1:1 (based on the initial seeding number of melanoma cells), followed by ring cloning. Polyclonal sublines (AR4, AR5) were derived using an E:T ratio of 1:2 and comprised resistant colonies from multiple wells. The initial acquired resistant sublines were expanded without T-cell treatments for three weeks, followed by continuous rounds of T-cell treatments until cryopreservation. To verify resistance, M486P and AR sublines were subjected to coculture with NY-ESO1 TCR-transduced T cells in 12-well plates at the indicated E:T ratios for 4 days. After coculture, plates were washed with PBS, and cell confluence was quantified with brightfield imaging using the IncuCyte SX5 with the 10x objective. Plates were fixed with 4% paraformaldehyde (Thermo) and stained with 0.1% crystal violet solution (Sigma).

To generate syngeneic murine melanomas with acquired resistance to ICB, we injected one million YUMM1.7ER cells subcutaneously on both flanks of male C57BL/6ROC mice. Once tumors reached volumes of 100–250 mm^3^, we started the ICB treatment. Anti-PD-1 (Leinco) and anti-CTLA-4 (Bio X Cell) were intraperitoneally injected twice a week (300 μg/mouse for each antibody for the first two weeks and then 200 μg/mouse). We selected tumors (AR1, AR2, and AR3) that regressed initially but later regrew for further analysis. We used a portion of the AR3 tumor, excised on day 148 after initiating anti-PD-1+anti-CTLA-4 therapy, for dissociation (kit and gentleMACS Octo Dissociator from Miltenyi Biotec) and derivation of a cell line (AR3cl). Within eight weeks of in vitro culture, the AR3cl cell line was used for experiments.

#### scWGS and analysis

We dissociated 3×3×3 mm flash-frozen YER or clinical tumor pieces in 4 ml NST-DAPI buffer using the gentleMACS Octo Dissociator (Miltenyi Biotec) with protocol 4c_nuclei. We sorted nuclei using the BD FACSAria II in which DAPI intensity was used to gate and harvest the diploid or aneuploid nuclei populations. Cultured M486 P line and AR sublines were digested by trypsin-EDTA and stained with 10 μg/ml Hoechst33342 (Thermo Fisher) and 5 μg/ml propidium iodide (BD) for 30 minutes on ice. 28,000 cells or nuclei per ml were dispensed into ICELL8 350v nanowell chip (Takara Bio) using the ICELL8 cx system (Takara Bio). We selected wells with single live cells or nuclei using the ICELL8 CellSelect Software (Takara Bio) and prepared WGS libraries following a published protocol.^65^ Briefly, the selected single cells or nuclei were lysed using a lysis buffer (30 mM Tris-HCl PH 8.0, 5% Tween 20, 0.5% TritonX-100, 1.36 AU/ml protease) for 30 minutes at 54.5°C. We then performed tagmentation using the Illumina Tagment DNA enzyme kit (Illumina) at 54.5°C for 12 mins. The reactions were stopped by dispensing neutralization buffer into the sample wells and incubation at 49.4°C for 30 minutes. 72 indexed PCR forward primers and 72 indexed reverse primers were used to barcode all the sample wells. PCR master mix was dispensed into each sample well, and single-cell whole genomes were amplified for 12 cycles. The PCR products were collected by the Collection Kit (Takara Bio) by centrifuging the chip facing downward at 3,000g for 10 minutes. The libraries were purified by 1.8x Ampure XP beads (Beckman), followed by Qubit and TapeStation QC. We sequenced the libraries using No-vaseq X Plus targeting 1 million reads (2×150 bp) per cell or nucleus.

FASTQ reads were aligned to the human reference genome hg19 or the mouse reference genome mm10 using bowtie2.^66^ PCR duplicates were subsequently removed using sambamba.^67^ We excluded cells or nuclei with excessive noise based on the following criteria: (1) low-quality mapping libraries (Q < 1), (2) read counts below 100K when the average read number per cell is around 1M, and (3) > 10% bins containing no mapped reads. We then counted aligned reads in variable bins (averaging 220 kb and normalized using GC content via lowess regression). Bin-wise ratios were calculated by dividing the bin counts by the mean bin count of each cell. Circular binary segmentation (CBS) function of the R package DNACopy^68^ was employed for segmentation. Low-quality CNVs were filtered out by using the k-nearest neighbor filtering function in CopyKit, with human cells/nuclei excluded if their average correlation with their five nearest neighbors was < 0.8 and mouse nuclei excluded if their average correlation with their four nearest neighbors was < 0.7 (doi: https://doi.org/10.1101/2022.03.09.483497).

To identify diploid cells, we calculated the coefficient of variation from the CBS segment ratio values for each cell. We initially simulated the expected coefficient of variation for 1,000 diploid cells using a normal distribution *N (0,0.01)*. By applying an expectation-maximization algorithm to the combined dataset of single-cell segment ratios and the simulated diploid coefficients of variation, we fitted a mixture of normal distributions using the *normalmixEM* function from the R package mixtools (v1.2.0). Cells/nuclei were classified as diploid and excluded from further analysis if their coefficients of variation exceeded 5 standard deviations from the mean of the distribution that included the simulated diploid dataset.

Segment ratios of individual cells were log2-transformed and subjected to dimension reduction via UMAP using the R package uwot (v0.1.16) with specific parameters (seed = 31, min dist = 0.1, n_neighbors = 20, distance = ‘manhattan’, spread = 3). Subsequently, we identified subclones using a density-based clustering algorithm (dbscan) in the R package dbscan (v1.1.12). Cells identified as noise by hdbscan were filtered out, and those failing to meet the minimum cell count criterion (*n* = 6) in each subclone were also excluded to mitigate clustering inaccuracies. We generated heatmaps using the R package ComplexHeatmap (v2.10.0).

To construct phylogenies, we first determined the total number of CNV events, computed as the count of CNV breakpoints in the consensus CNV profile of each subclone. The length of CNV events was estimated based on the number of bins between each pair of breakpoints, excluding events with a length of ≤ 1 bin. Ploidy estimation for each sample was conducted using DAPI signals. Specifically, we employed the formula 2 × (median DAPI intensity of A peak / median DAPI intensity of D peak) if the majority of sequenced cells/nuclei originated from the aneuploid peak. We then derived the subclonal consensus integer profiles by computing the median of every integer CN across all single cells/nuclei assigned to the same subclone, rounded to the nearest integer. The phylogenetic trees of subclones were generated using MEDICC2,^53^ utilizing minimum event distances. To root the tree, a diploid cell with a copy number of 2 was introduced as the root node. We visualized trees using the R package ggtree (v3.11.0).^69^

We culled CNVs specific to M486 AR3 and AR5 (versus M486 P), YER AR3 (versus YER P/vehicle 1), and Pt18 recurrence (versus Pt18 baseline) and CNV-affected genes. We then categorized CNV-affected genes as (i) private to a subset of AR/recurrence-dominant subclones (defined as > 50% of cells from the AR/recurrence sample(s)), or (ii) preexisting in a subset of P/baseline-dominant subclones (defined as > 50% of cells from the P or baseline sample). We first calculated the median copy-number ratio (hereafter referred to as “ratio”) for each gene for all the cells/nuclei of each subclone. For these ratios, we calculated the coefficient of variation (CV) across all baseline-dominant subclones and selected genes whose ratios’ CVs were < 0.18 for further analysis. We defined an AR/recurrence-specific amplified gene if one or more AR-dominant subclone(s) exhibited a ratio exceeding the maximum ratio observed among baseline-dominant subclones by more than 0.09. Similarly, we defined an AR/recurrence-specific deleted gene if one or more AR-dominant subclone(s) exhibited a ratio lower than the minimum ratio observed among baseline-dominant subclones by more than 0.09. To identify AR/recurrence-specific, amplified or deleted genes that preexisted in one or more P/base-line-dominant subclones, we first selected genes whose ratios’ CVs were > 0.18 across all P/baseline-dominant subclones. For these genes, we then calculated z-scores of the ratios for every P/baseline-dominant subclone and classified P/baseline-dominant subclone(s) with absolute z-scores > 1 as candidate(s) harboring preexisting AR/recurrence-specific amplified or deleted genes. Next, two criteria must be met to identify true preexisting P/baseline-dominant subclones. First, for every P/baseline-preexisting amplified or deleted gene, any AR/recurrence-dominant subclone must display a ratio for that gene exceeding the maximum ratio by > 0.09 or below the minimum ratio by > 0.09, respectively, for the same gene observed across all the non-candidate P/baseline-dominant subclones. Second, for every P/baseline-preexisting amplified or deleted gene, any candidate preexisting P/baseline-dominant subclone must display a ratio for that gene exceeding the maximum ratio by > 0.09 or below the minimum ratio by > 0.09, respectively, for the same gene observed across all the non-candidate P/baseline-dominant subclones.

We further culled all AR/recurrence-specific CNV-affected genes (inclusive of those private to a subset of AR/recurrence-dominant subclones and preexisting in a subset of P/baseline-dominant subclones) (Table S7) with roles in programmed cell death (including apoptosis, ferroptosis, etc.) and immune regulation. Genes with such functions were compiled from 11 gene sets from the following databases: Gene Ontology Biological Processes (Activation of Innate Immune Response, Adaptive Immune Response, Apoptotic Signaling Pathway), WikiPathways (Cancer Immunotherapy by PD-1 Blockade, Cancer Immunotherapy by CTLA-4 Blockade), Hallmark (Apoptosis), and Kyoto Encyclopedia of Genes and Genomes (Apoptosis, PD-L1 Expression and PD-1 Checkpoint Pathway in Cancer, Antigen Processing and Presentation, Ferroptosis, Chemokine Signaling Pathway). We annotated all CNV-affected genes with the weights of concordant evidence from CRISPR-Cas9 screens (Table S3). Thus, an AR/recurrence-specific, amplified gene can be functionalized by supportive evidence as a sensitizer gene; an AR/recurrence-specific, deleted gene can be functionalized by supportive evidence as a resister gene. Lastly, if CNV-affected genes were identified as amplified or deleted specifically in distinct AR/recurrence-dominant subclones, we denoted this using a semicolon (;) to separate subclones of these two classifications.

#### Enrichment analysis

For the current clinical cohort, we used Metascape^70^ (REACTOME gene sets) for enrichment analysis of recurrent (≥ 4 patients) CNV-affected genes. For the validation clinical cohort, we used processed CNV segments from pre-and-post (14 pre, 16 post) melanomas of 14 patients and identified recurrent (> 2 patients) CNV genes.^71^ We performed enrichment analysis via DAVID (Gene Ontology Biological Process terms) for overlapping genes between the current or validation clinical cohort (bulk WES) and the CRISPR-Cas9 screen hits, between the five M486 AR sublines and the M486 P line (bulk WGS), between the three YER AR tumors and the vehicle-treated P tumor (bulk WGS), and between the YER AR3 tumor and the vehicle-treated parental tumor (scWGS).

#### Western blots

We measured protein levels after lysis of cells in RIPA buffer (Thermo Fisher) with protease (Thermo Fisher) and phosphatase (Thermo Fisher) inhibitor cocktails. We determined protein concentrations by the BCA protein assay (Thermo Fisher). Primary antibodies used are as follows: TUBULIN (CST), BAX (CST), FAS (Thermo Fisher), FLAG (CST), P53 (CST), TNFR1 (CST), BAD (CST), TAP1 (CST), TAP2 (CST), and IFNGR1 (CST). All antibodies were diluted 1:1,000.

#### BH3 profiling

We washed trypsinized cultured cells with cold PBS, resuspended them in iBH3 buffer (150 mM D-mannitol, 5 mM succinate, 10 mM HEPES, 1 mM EGTA, 1 mM EDTA, 150 mM KCl, 0.25%(w/v) pluronic F68, 0.1% BSA, pH 7.4), and plated cells at 4 × 10^4^ cells/well in v-bottom 96-well plates. We then treated the samples with 0.001% digitonin (Promega) and the indicated concentrations of BH3-mimetic peptides. Treatment with ALA (alamethicin) served as a positive control. We incubated the plates at room temperature for 50 min, fixed the cells with 4% paraformaldehyde in PBS for 10 min, and neutralized the fixation with 1.5 M Tris base for 5 min. We stained the samples with anti-cytochrome c (BioLegend) and DAPI at 4°C overnight and analyzed cytochrome c retention by flow cytometry (Attune NxT, ThermoFisher). Mitochondrial cytochrome c release was calculated using the formula 100%–100% x (cytochrome c retention by BH3 peptide–cytochrome c retention by ALA)/(cytochrome c retention by DMSO–cytochrome c retention by ALA). All BH3 peptides were synthesized by Biosynth.

### QUANTIFICATION AND STATISTICAL ANALYSIS

We compared two independent groups using the Student’s t test and made multiple comparisons using the one-way ANOVA test. The two-way ANOVA test was used to compare across cell or tumor growth curves. We assessed the co-occurrence or association between two events using the Fisher’s exact test. Fisher’s exact test was utilized to assess the co-occurrence or association between two events. All details related to statistical analysis can be found in figure legends. All analyses were performed using R.4.02, Python 3.8.0, Python 2.7.17 and GraphPad Prism 10. The *P* values were represented as follows: ns or not significant, *p < 0.05, **p < 0.01, ***p < 0.001.

## Supplementary Material

1

2

3

4

5

6

7

8

Supplemental information can be found online at https://doi.org/10.1016/j.immuni.2025.10.001.

## Figures and Tables

**Table:** KEY RESOURCES TABLE

REAGENT or RESOURCE	SOURCE	IDENTIFIER

Antibodies		

beta-2 Microglobulin Monoclonal Antibody (B2M-02)	Invitrogen	Cat# MA1–19413; RRID: AB_1070703
GSDMC Polyclonal Antibody	Invitrogen	Cat# PA5–116594; RRID: AB_2901225
Fas Recombinant Rabbit Monoclonal Antibody (JJ0942)	Invitrogen	Cat# MA5–32489; RRID: AB_2809766
DFFB Polyclonal Antibody	Invitrogen	Cat# PA5–115114; RRID: AB_2899750
Caspase 9 Monoclonal Antibody (LAP6 96–2-22)	Invitrogen	Cat# MA1–16842; RRID: AB_568482
Bad Antibody	Cell Signaling Technology	Cat# 9292; RRID: AB_331419
alpha-Tubulin Antibody	Cell Signaling Technology	Cat# 2144; RRID: AB_2210548
Bax (E4U1V) Rabbit mAb	Cell Signaling Technology	Cat# 41162; RRID: AB_2924730
DYKDDDDK Tag (D6W5B) Rabbit mAb	Cell Signaling Technology	Cat# 14793; RRID: AB_2572291
p53 (7F5) Rabbit mAb	Cell Signaling Technology	Cat# 2527; RRID: AB_10695803
TNF-R1 (C25C1) Rabbit mAb	Cell Signaling Technology	Cat# 3736; RRID: AB_2241018
TAP1 (E4T4F) Rabbit mAb	Cell Signaling Technology	Cat# 49671
TAP2 (E8G5I) Rabbit mAb	Cell Signaling Technology	Cat# 25657
IFNGR1 Antibody	Cell Signaling Technology	Cat# 34808; RRID: AB_2799061
FITC anti-human CD3 Antibody	BioLegend	Cat# 317306; RRID: AB_571907
PE anti-human TCR Vβ13.1 Antibody	BioLegend	Cat# 362410; RRID: AB_2750159
Goat anti-Mouse IgG (H+L) Highly Cross-Adsorbed Secondary Antibody, Alexa Fluor™ Plus 488	Invitrogen	Cat# A32723; RRID: AB_2633275
Goat anti-Rabbit IgG (H+L) Highly Cross-Adsorbed Secondary Antibody, Alexa Fluor™ 555	Invitrogen	Cat# A-21429; RRID: AB_2535850

Bacterial and virus strains		

NEB^®^ Stable Competent E. coli (High Efficiency)	New England Biolabs	Cat# C3040H

Biological samples		

Tumor and patient-matched normal tissues	University of California, Los Angeles, Sutter Health, and the Vanderbilt-Ingram Cancer Center	See Table S1 for details
Peripheral blood mononuclear cells (PBMCs)	UCLA virology core laboratory	N/A
Mouse syngeneic tumors	Dr. Roger Lo	N/A

Chemicals, peptides, and recombinant proteins		

Anti-Mouse CD279 (PD-1) (Clone RMP1–14)	Leinco Tech	Cat# P362
InVivoMAb anti-mouse CTLA-4 (CD152)	BioXcell	Cat# BE0131
Venetoclax	TargetMol	Cat# T2119
PEG300	MedChemExpress	Cat# HY-Y0873
Antigen Retrieval Buffer	Abcam	Cat# ab93684
DAPI	MilliporeSigma	Cat# D9542
Molecular Probes^™^ ProLong^™^ Diamond Antifade Mountant	Thermo Fisher Scientific	Cat# P36965
BioT	Bioland Scientific	Cat# B01–00
Sodium butyrate	Thermo Fisher Scientific	Cat# A11079.36
HEPES	Thermo Fisher Scientific	Cat# 15630080
Recombinant human IL-2	Miltenyi Biotec	Cat# 130–097-748
Dynabeads^™^ Human T-Activator CD3/ CD28 for T Cell Expansion and Activation	Gibco	Cat# 11161D
RetroNectin	Takara	Cat# T110A
Cryostor CS10 cryopreservation medium	Biolife Solutions	Cat# 210102
G418 disulfate salt	MilliporeSigma	Cat# A1720–1G
Blasticidin S HCl	Gibco	Cat# R21001
Puromycin	MilliporeSigma	Cat# P8833–25MG
Hygromycin B	Thermo Fisher Scientific	Cat# AAJ6068103
Polybrene Infection / Transfection Reagent	MilliporeSigma	Cat# TR-1003-G
RIPA Lysis and Extraction Buffer	Thermo Fisher Scientific	Cat# 89901
Halt^™^ Phosphatase Inhibitor Single-Use Cocktail	Thermo Fisher Scientific	Cat# 78428
Pierce^™^ BCA Protein Assay Kits	Thermo Fisher Scientific	Cat# 23227
Tween 80	Thermo Fisher Scientific	Cat# BP338–500
4% Paraformaldehyde in PBS	Thermo Fisher Scientific	Cat# J61899.AP
Crystal violet solution	MilliporeSigma	Cat# V5256
Tris-HCl (PH7.4)	MilliporeSigma	Cat# T2194
Tween 20	Andwin Scientific	Cat# NC9022994
Nonidet P40 Substitute	MilliporeSigma	Cat# 74385
NaCl	Invitrogen	Cat# AM9760G
MgCl2	Invitrogen	Cat# AM9530G
Triton X-100	Thermo Fisher Scientific	Cat# ICN19485483
QIAGEN Protease (30 AU)	Qiagen	Cat# 19157
0.5M EDTA	Thermo Fisher Scientific	Cat# R1021
Xylene	Fisher Scientific	Cat# X5–1
Ethyl Alcohol	Sigma-Aldrich	Cat# E7023

Critical commercial assays		

QIAGEN AllPrep DNA/RNA Mini Kit	Qiagen	Cat# 80204
QIAGEN QIAamp DNA FFPE Tissue Kit	Qiagen	Cat# 56404
QIAGEN FlexiGene DNA Kit	Qiagen	Cat# 51206
Pan T Cell Isolation Kit	Miltenyi Biotec	Cat# 130–096-535
Tumor Dissociation Kit, mouse	Miltenyi Biotec	Cat# 130–096-730
FFPE Tissue Dissociation Kit	Miltenyi Biotec	Cat# 130–118-052
Illumina Tagment DNA Enzyme and Buffer Large Kit	Illumina	Cat# 20034198
KAPA HotStart PCR Kit, with dNTPs	Roche	Cat# KK2502
Qubit dsDNA HS (High Sensitivity) Assay Kit	Invitrogen	Cat# Q32851
Qubit dsDNA BR (Broad-Range) Assay Kit	Invitrogen	Cat# Q32850

Deposited data		

WES	This paper	European Genome-phenome Archive (EGA): EGAS50000001055
WGS	This paper	EGA: EGAS50000001055
WES	Zaretsky et al.^15^	SRA: SRP076315
WES	Hugo et al.^11^	SRA: SRP067938, SRP090294
WES	Liu et al., ^13^ Hugo et al.,^44^ Moriceau et al.,^45^ Shi et al.^46^	SRA: SRP049746
Copy number profiles of TCGA-SKCM cohort	TCGA, PanCancer Atlas	https://www.cbioportal.org/
scWGS	This paper	dbGaP study accession: phs004102.v1

Experimental models: Cell lines		

A375	Dr. Roger S. Lo	N/A
M257	Dr. Roger S. Lo	N/A
M407	Dr. Roger S. Lo	N/A
M486	Dr. Antoni Ribas	PMID: 36890230
M486 AR1–AR5	Dr. Roger S. Lo	This paper
YUMM1.7ER	Dr. Roger S. Lo	Wang et al.^52^
YUMM1.7ER AR3cl	Dr. Roger S. Lo	This paper

Experimental models: Organisms/strains		

Mouse: C57BL/6ROC	UCLA: Radiation-Oncology Colony	C57BL/6J/NROC

Recombinant DNA		

mCherry-NLS	Addgene	Cat# 39319
H2B-GFP	Addgene	Cat# 11680
DFFA-CR	Dr. Stephen Elledge	PMID: 31398327
human BAX	UCLA Molecular Screening Shared Resource	N/A
human BAD	UCLA Molecular Screening Shared Resource	N/A
human P53	UCLA Molecular Screening Shared Resource	N/A
human TNFR1	UCLA Molecular Screening Shared Resource	N/A
Mouse Bad	Dr. Roger S. Lo	This paper
Mouse Fas	Dr. Roger S. Lo	This paper

Software and algorithms		

GraphPad Prism	https://www.graphpad.com	Version: 10
ImageJ	https://imagej.net/ij/	Version: 1.54d
ICELL8 cx CellSelect	Takara	Version: 2.6.51.0
ICELL8 cx CELLSTUDIO	Takara	Version: 2.6.42.0
R software	CRAN	Version: 4.0.2
BWA	http://bio-bwa.sourceforge.net/	Version: 0.7.17
Picard	https://broadinstitute.github.io/picard/	Version: 1.141
gatk	https://gatk.broadinstitute.org/hc/en-us	Version: 3.8
Samtools	http://www.htslib.org/	Version: 1.19.2
MuTect	https://github.com/broadinstitute/mutect	Version: 1.1.7
VarScan2	http://varscan.sourceforge.net/	Version: 2.4.3
Oncotator	https://github.com/broadinstitute/oncotator	Version: 1.9.9.0
Sequenza	https://sequenzatools.bitbucket.io/	Version: 2.1.2
PHYLIP	https://phylipweb.github.io/phylip/	Version: 3.698
Strelka2	https://github.com/Illumina/strelka	Version: 2.9.2
SigProfilerAssignment	https://github.com/AlexandrovLab/SigProfilerAssignment	Version: 0.0.1
Metascape	https://metascape.org/	Version: v3.5.20240101
MutSig2CV	https://github.com/getzlab/MutSig2CV	Version. 3.1
maftools	https://github.com/PoisonAlien/maftools	Version: 2.6.05
MEDICC2	https://bitbucket.org/schwarzlab/medicc2	Version: 1.0.2
Bowtie2	https://bowtie-bio.sourceforge.net/bowtie2/	Version: 2.4.2
Sambamba	https://lomereiter.github.io/sambamba/	Version: 0.8.0
DNACopy	https://bioconductor.org/packages/release/bioc/html/DNAcopy.html	Version: 1.78.0
CopyKit	https://github.com/navinlabcode/copykit	Version: 0.1.2
mixtools	https://github.com/dsy109/mixtools	Version: 1.2.0
uwot	https://github.com/jlmelville/uwot	Version: 0.1.16
dbscan	https://github.com/mhahsler/dbscan	Version: 1.1.12
ComplexHeatmap	https://github.com/jokergoo/ComplexHeatmap	Version: 2.10.0
ggtree	https://github.com/YuLab-SMU/ggtree	Version: 3.11.0
ANNOVAR	https://annovar.openbioinformatics.org/	N/A

## Highlights

- Acquired-resistant melanomas amplify anti-apoptotic and/or delete pro-apoptotic genes
- Models of acquired ICI resistance recapitulate apoptotic evasion due to CNVs
- Preexisting and *de novo* resistance-driver CNVs occur commonly or privately in subclones
- Lowering the apoptotic threshold salvages ICI sensitivity and prevents relapse
