# Supplementary material for: Genomic copy-number variants drive apoptotic evasion underlying acquired resistance to immune checkpoint inhibitors
Source: Immunity. Author manuscript; Available in PMC 2025 Dec 10. (PMC12693071; doi:10.1016/j.immuni.2025.10.001)
Supplement: 1 [file NIHMS2120882-supplement-1.pdf]

Figure S1

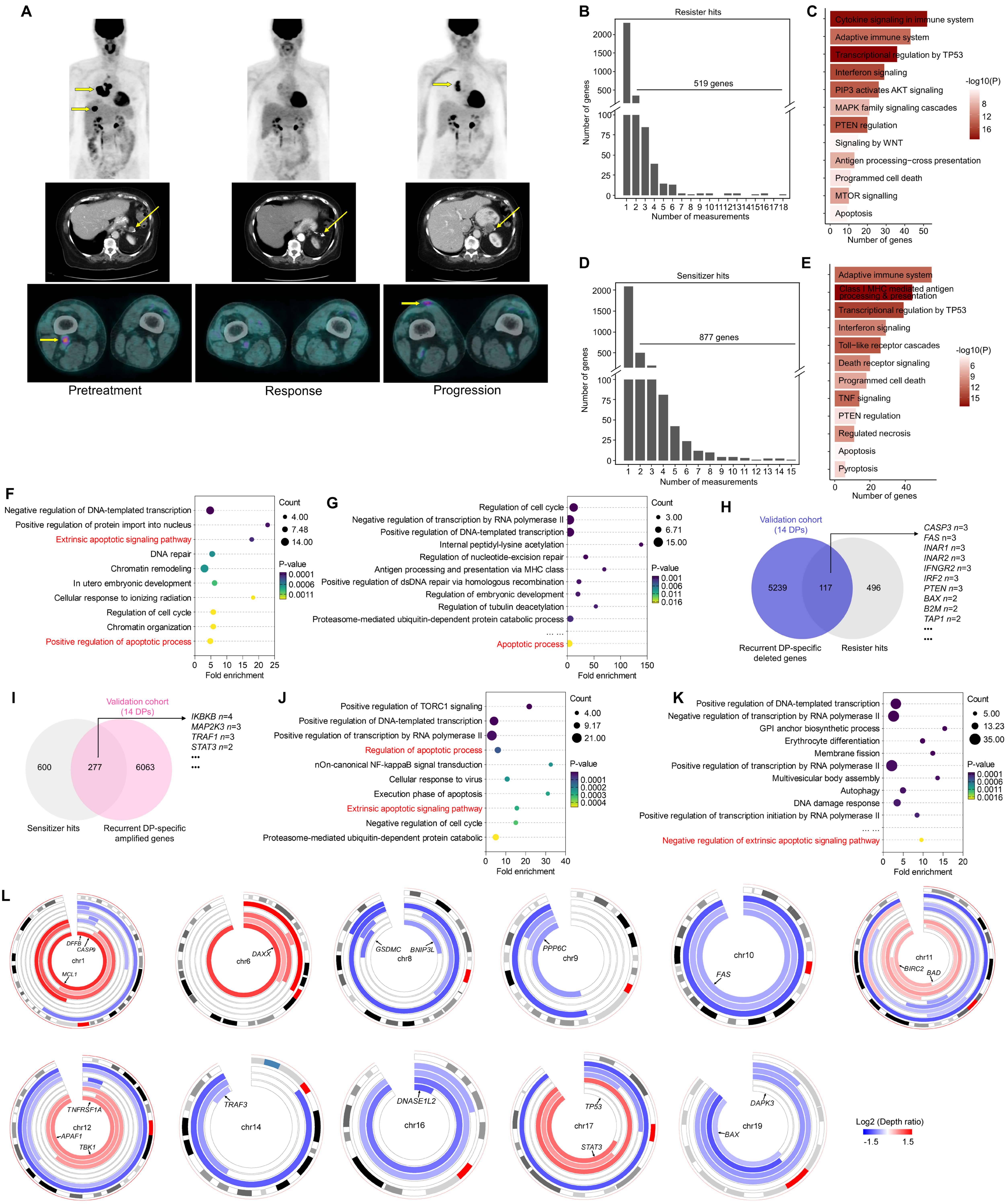

A
Pretreatment
Response
Progression
B
Resister hits
519 genes
Number of genes
Number of measurements
C
Cytokine signaling in immune system
Adaptive immune system
Transcriptional regulation by TP53
Interferon signaling
PIP3 activates AKT signaling
MAPK family signaling cascades
PTEN regulation
Signaling by WNT
Antigen processing−cross presentation
Programmed cell death
MTOR signalling
Apoptosis
-log10(P)
Number of genes
D
Sensitizer hits
877 genes
Number of genes
Number of measurements
E
Adaptive immune system
Class I MHC mediated antigen processing & presentation
Transcriptional regulation by TP53
Interferon signaling
Toll−like receptor cascades
Death receptor signaling
Programmed cell death
TNF signaling
PTEN regulation
Regulated necrosis
Apoptosis
Pyroptosis
-log10(P)
Number of genes
F
Negative regulation of DNA-templated transcription
Positive regulation of protein import into nucleus
Extrinsic apoptotic signaling pathway
DNA repair
Chromatin remodeling
In utero embryonic development
Cellular response to ionizing radiation
Regulation of cell cycle
Chromatin organization
Positive regulation of apoptotic process
Count
4.00
7.48
14.00
P-value
0.0001
0.0006
0.0011
Fold enrichment
G
Regulation of cell cycle
Negative regulation of transcription by RNA polymerase II
Positive regulation of DNA-templated transcription
Internal peptidyl-lysine acetylation
Regulation of nucleotide-excision repair
Antigen processing and presentation via MHC class
Positive regulation of dsDNA repair via homologous recombination
Regulation of embryonic development
Regulation of tubulin deacetylation
Proteasome-mediated ubiquitin-dependent protein catabolic process
... ...
Apoptotic process
Count
3.00
6.71
15.00
P-value
0.001
0.006
0.011
0.016
Fold enrichment
H
Validation cohort
(14 DPs)
5239
117
496
Recurrent DP-specific deleted genes
Resister hits
CASP3 n=3
FAS n=3
INAR1 n=3
INAR2 n=3
IFNGR2 n=3
IRF2 n=3
PTEN n=3
BAX n=2
B2M n=2
TAP1 n=2
I
Validation cohort
(14 DPs)
600
277
6063
Sensitizer hits
Recurrent DP-specific amplified genes
IKBKB n=4
MAP2K3 n=3
TRAF1 n=3
STAT3 n=2
J
Positive regulation of TORC1 signaling
Positive regulation of DNA-templated transcription
Positive regulation of transcription by RNA polymerase II
Regulation of apoptotic process
nOn-canonical NF-kappaB signal transduction
Cellular response to virus
Execution phase of apoptosis
Extrinsic apoptotic signaling pathway
Negative regulation of cell cycle
Proteasome-mediated ubiquitin-dependent protein catabolic
Count
4.00
9.17
21.00
P-value
0.0001
0.0002
0.0003
0.0004
Fold enrichment
K
Positive regulation of DNA-templated transcription
Negative regulation of transcription by RNA polymerase II
GPI anchor biosynthetic process
Erythrocyte differentiation
Membrane fission
Positive regulation of transcription by RNA polymerase II
Multivesicular body assembly
Autophagy
DNA damage response
Positive regulation of transcription initiation by RNA polymerase II
... ...
Negative regulation of extrinsic apoptotic signaling pathway
Count
5.00
13.23
35.00
P-value
0.0001
0.0006
0.0011
0.0016
Fold enrichment
L
chr1
DFFB
CASP9
MCL1
chr6
DAXX
chr8
GSDMC
BNIP3L
chr9
PPP6C
chr10
FAS
chr11
BIRC2
BAD
chr12
TNFRSF1A
APAF1
TBK1
chr14
TRAF3
chr16
DNASE1L2
chr17
TP53
STAT3
chr19
DAPK3
BAX
Log2 (Depth ratio)
-1.5
1.5

Figure S1. Analyses of DP-specific CNV deleted and amplified genes plus CRISPR-Cas9 screen-derived resister and sensitizer genes, related to Figure 1 and Table S1–S3

(A) Examples of in situ (top, middle) and de novo (bottom) acquired-resistant melanomas to ICI therapy. (Top) PET-CT images at the indicated timepoints for Pt#11 (50-year-old female) who presented with cutaneous melanoma metastatic to the right mediastinum/lung and liver and underwent therapy with ipilimumab plus nivolumab. Treatment resulted in a complete metabolic response (center PET-CT at ~9 months into treatment). At ~2.5 years after treatment, a mediastinal mass emerged during active treatment. (Middle) CT images at the indicated timepoints for Pt#15 (73-year-old female) who presented with cutaneous melanoma metastatic to the splenic soft tissue bed. She started pembrolizumab treatment with initial clinical response (center CT at 6 months into treatment). At ~2 years into treatment, a mass at the splenic soft tissue bed emerged during active treatment. (Bottom) PET-CT images for Pt#8 (60-year-old male) who presented with large acral primary melanoma and popliteal lymph node metastasis and underwent therapy with ipilimumab plus nivolumab, which resulted in a near complete response. At ~11 months into treatment, he developed a metastatic acral melanoma to the subcutaneous skin.

(B) The numbers of resister genes per numbers of independent CRISPR-Cas9 screen measurements from 23 publications.

(C) Significantly enriched immune-, cell death-, innate ICI resistance-related Reactome gene sets of 519 resister gene hits in B.

(D) As in B, except for sensitizer genes.

(E) As in C, except for 877 sensitizer genes in D.

(F and G) The top 10 enriched Gene Ontology terms of overlapping genes from Figure 1D (F) and 1E (G, top 10 plus term ranked #23).

(H) Based on WES data of the validation cohort (*n* = 14 patients, 14 baseline and 16 DP tumors), the numbers of recurrent (≥ 2 of 14 patients), DP-specific (patient-matched) deleted genes (left circle), resister genes (right circle), and overlapping genes (select genes shown with the numbers of patients affected).

(I) As in H, except for recurrent, DP-specific amplified genes (right circle) and sensitizer genes (left circle).

(J and K) As in F and G, except for the validation cohort (K, top 10 plus term ranked #27).

(L) Circos plots showing DP-specific overlapping deletions and amplifications harboring apoptotic genes. Outermost layer, chromosome regions. Each inner layer represents a distinct DP tumor.

Figure S2

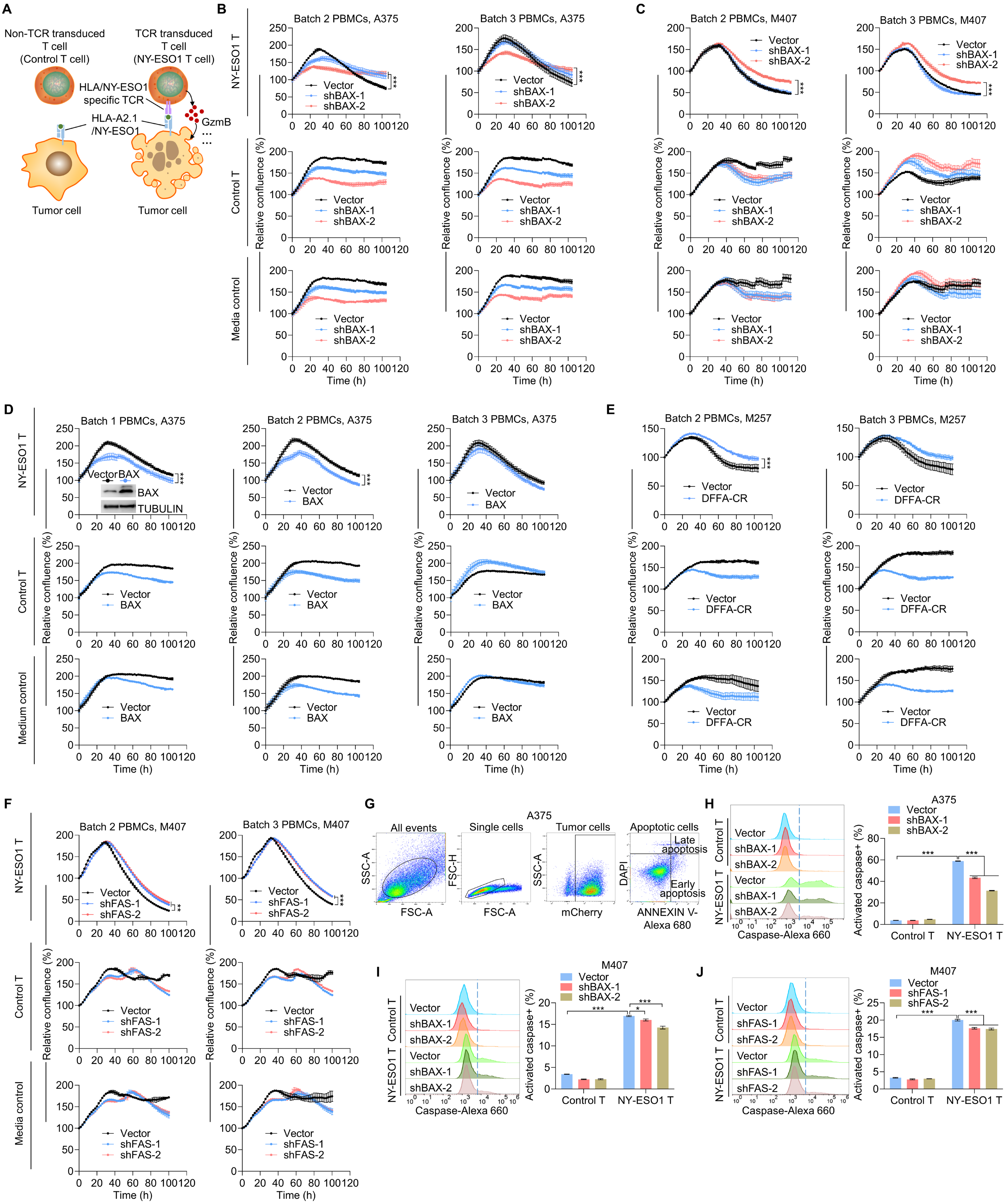

A
Non-TCR transduced T cell (Control T cell)
TCR transduced T cell (NY-ESO1 T cell)
HLA/NY-ESO1 specific TCR
HLA-A2.1 /NY-ESO1
GzmB
Tumor cell
Tumor cell
B
Batch 2 PBMCs, A375
Batch 3 PBMCs, A375
C
Batch 2 PBMCs, M407
Batch 3 PBMCs, M407
NY-ESO1 T
Control T
Media control
Relative confluence (%)
Time (h)
Vector
shBAX-1
shBAX-2
***
D
Batch 1 PBMCs, A375
Batch 2 PBMCs, A375
Batch 3 PBMCs, A375
Vector BAX
BAX
TUBULIN
Vector
BAX
Medium control
E
Batch 2 PBMCs, M257
Batch 3 PBMCs, M257
Vector
DFFA-CR
F
Batch 2 PBMCs, M407
Batch 3 PBMCs, M407
Vector
shFAS-1
shFAS-2
**
G
A375
All events
Single cells
Tumor cells
Apoptotic cells
SSC-A
FSC-A
FSC-H
mCherry
DAPI
Late apoptosis
Early apoptosis
ANNEXIN V-Alexa 680
H
A375
Vector
shBAX-1
shBAX-2
Caspase-Alexa 660
Activated caspase+ (%)
Control T
NY-ESO1 T
I
M407
*
J
M407
Vector
shFAS-1
shFAS-2

Figure S2. Coculture analyses of individual DP-specific and deleted pro-apoptotic genes, related to Figure 1

(A) Schematic of the coculture assay.

(B and C) As in Figure 1I and 1J, except with T cells from two additional donors.

(D) mCherry$^+$ A375 cells ± *BAX* overexpression (western blots with TUBULIN as loading control) and their growth patterns in coculture with HLA-/antigen-specific TCR-transduced primary T cells (top, E:T ratio of 1:3), with control or non-TCR-transduced primary T cells (middle, E:T ratio of 1:3), and without primary T cells but with primary T cell media only (bottom). Data shown for cocultures using primary T cells from three batches of PBMCs.

(E and F) As in Figure 1L and 1M, except with T cells from two additional donors.

(G) Example (from A375) of flow cytometry gating strategy for early and late apoptosis detection in Figure 1N–1P.

(H–J) Detection of pan-activated caspases in indicated cell lines, transduced stably with empty vector, shBAX (H, I), or shFAS (J), after cocultures for two days with non-TCR-transduced (control) or HLA-/antigen-specific TCR-transduced primary T cells (E:T ratios, 1:3 in H and 1:5 in I and J). Data represent 2–3 independent experiments for each T cell donor. Results (B–J) shown as mean (triplicates) ± SEM; *P* values, two-way (B–F) or one-way (H–J) ANOVA tests.

Figure S3

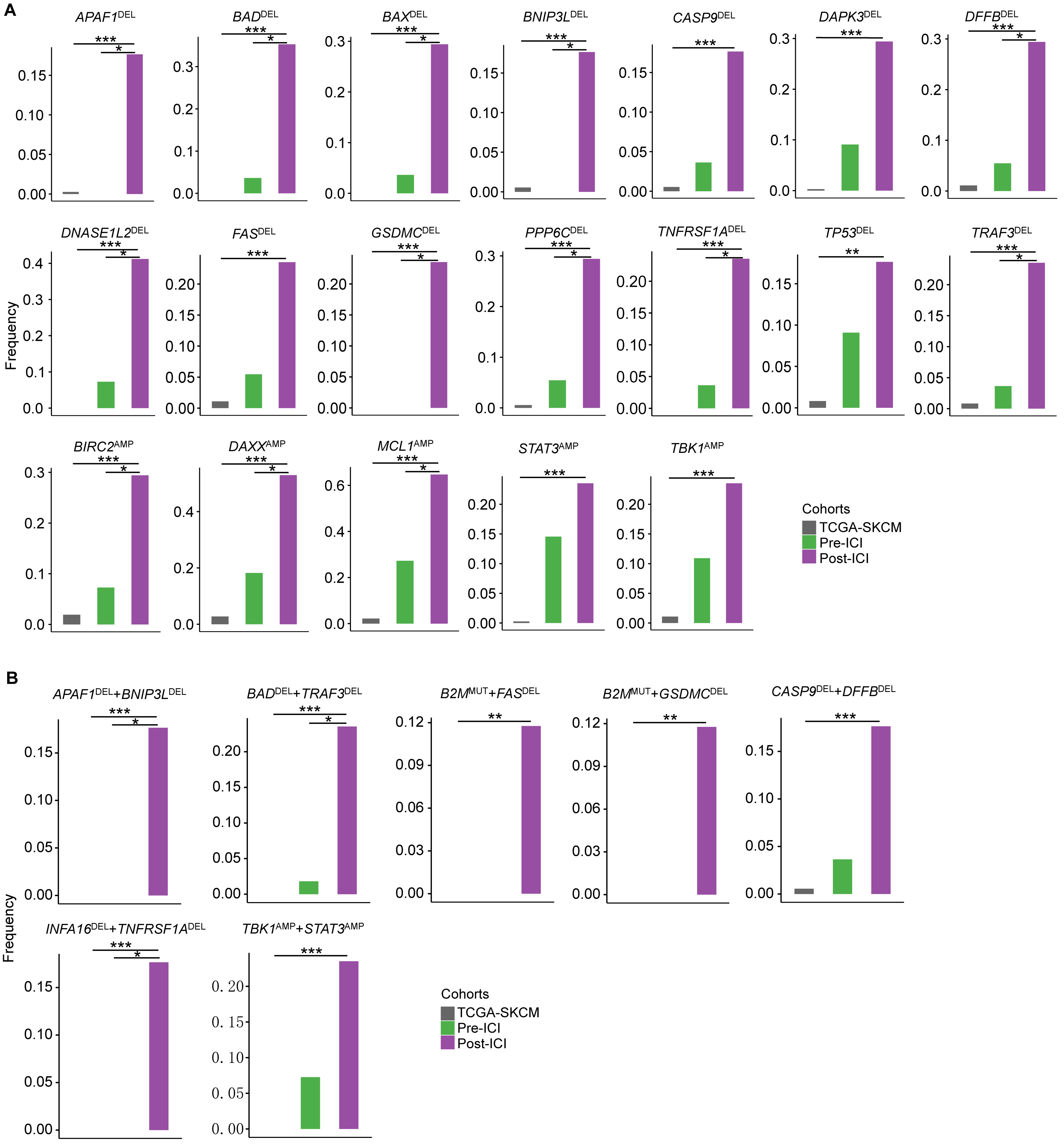

Figure S3. Frequencies of DP-specific gene mutations or co-mutations in ICI-naïve (versus the current DP) melanomas, related to Figure 2, Table S5 and S6

(A) Frequencies of deletions in pro-apoptotic genes or amplifications in anti-apoptotic genes in distinct cutaneous melanoma cohorts: (i) TCGA-SKCM (*n* = 367 melanomas from 367 patients), (ii) ICI-naive melanomas (*n* = 56 tumors from 55 patients), and (iii) post-ICI or DP melanomas from the current cohort (*n* = 20 tumors from 17 patients). *P* values of pairwise comparisons (post-ICI to TCGA-SKCM or ICI-naive), Fisher's exact test.

(B) As in A, except for frequencies of significant co-occurring gene pairs in Figure 2A.

Figure S4

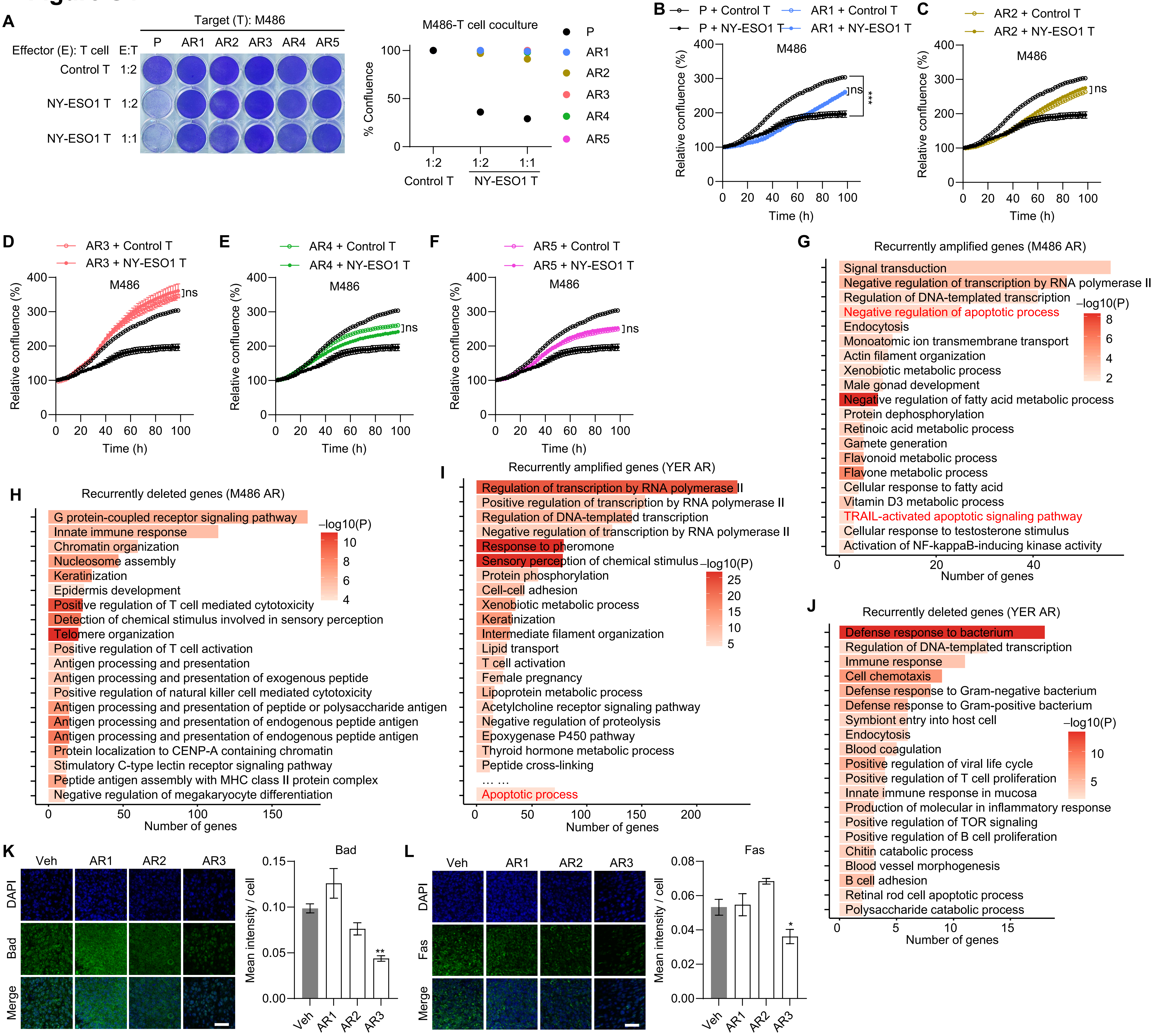

Figure S4. Characterization of human and murine melanoma AR models, related to Figure 3 and 4

(A) Crystal violet staining (left) and IncuCyte quantification of bright-field cell confluence (right) of the M486 P line and AR sublines, following a 4-day coculture with non-transduced (control) (E:T ratio of 1:2) or indicated E:T ratios of HLA-/antigen-specific primary T cells.

(B–F) As in Figure 3B–3F, except E:T ratio at 1:2. Mean ± SEM, *P* values by the two-way ANOVA test.

(G and H) The top 20 enriched gene ontology terms of recurrent (5/5 M486 AR sublines), AR-specific amplified (G) or deleted (H) genes.

(I and J) As in G and H, except recurrence defined as ≥ 2/3 YER AR tumors (I, top 20 plus term ranked #40).

(K and L) Immunofluorescent staining of Bad (K) and Fas (L) in isogenic YER P (vehicle) and AR3 (on ICIs) tumors. Representative images (left; ruler, 50 mm) and quantifications (right) of the mean fluorescent intensities from 5 or 6 fields. Mean ± SEM; *P* values, one-way ANOVA followed by Tukey's multiple comparison test.

Figure S5

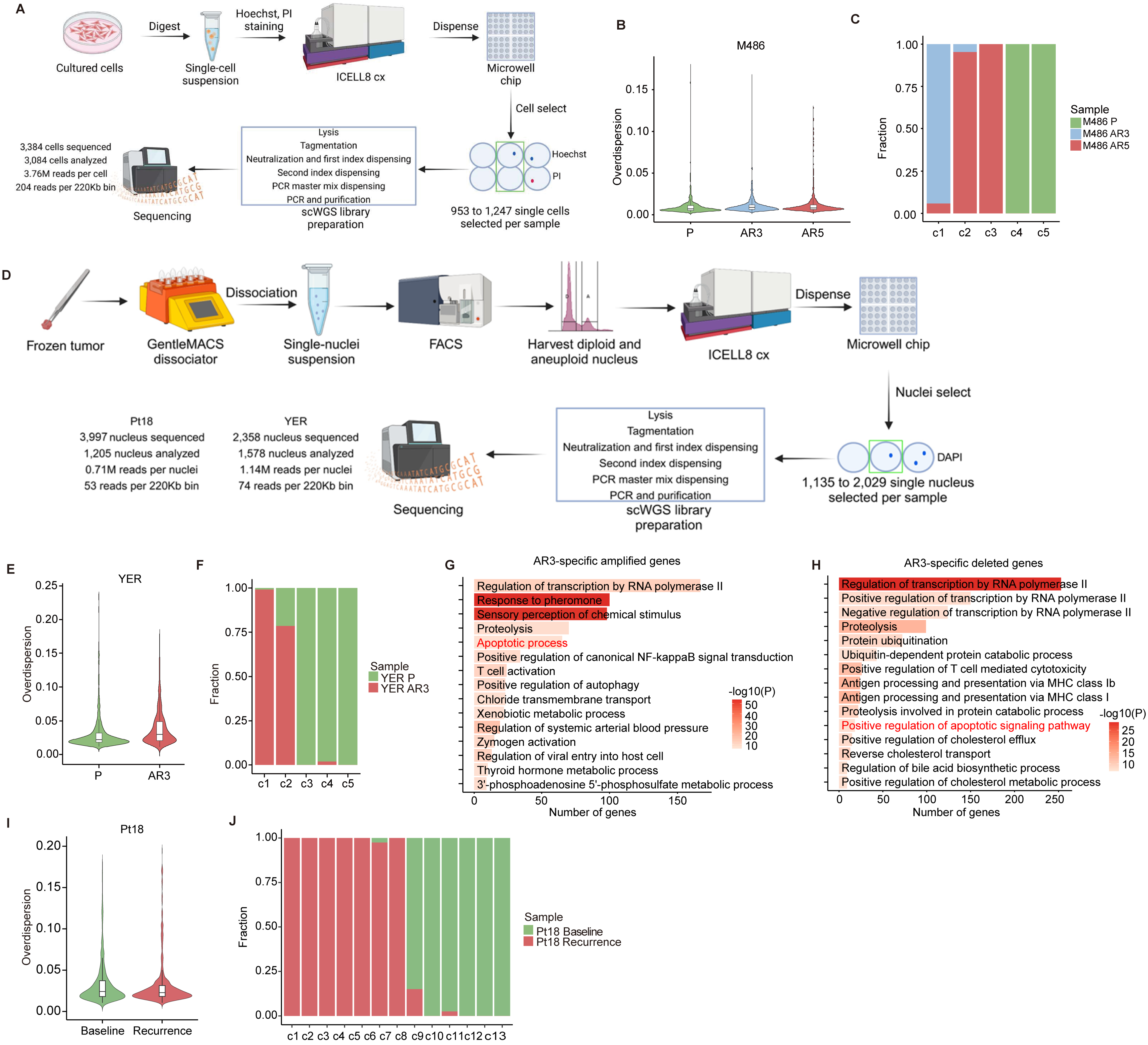

Figure S5. Workflow and analyses of scWGS data, related to Figure 5 and Table S7

(A) Schematic of scWGS workflow using cultured cells.

(B) Overdispersion of bin counts computed from scWGS data of indicated cell lines.

(C) Proportions of indicated cell lines contributing to the indicated subclones.

(D) As in A, except using frozen murine or clinical melanoma tumors.

(E and F) As in B and C, except for indicated murine tumors.

(G and H) The top 15 enriched gene ontology terms of AR3-specific, amplified (G) or deleted (H) genes.

(I and J) As in B and C, except for indicated clinical tumors.

Figure S6

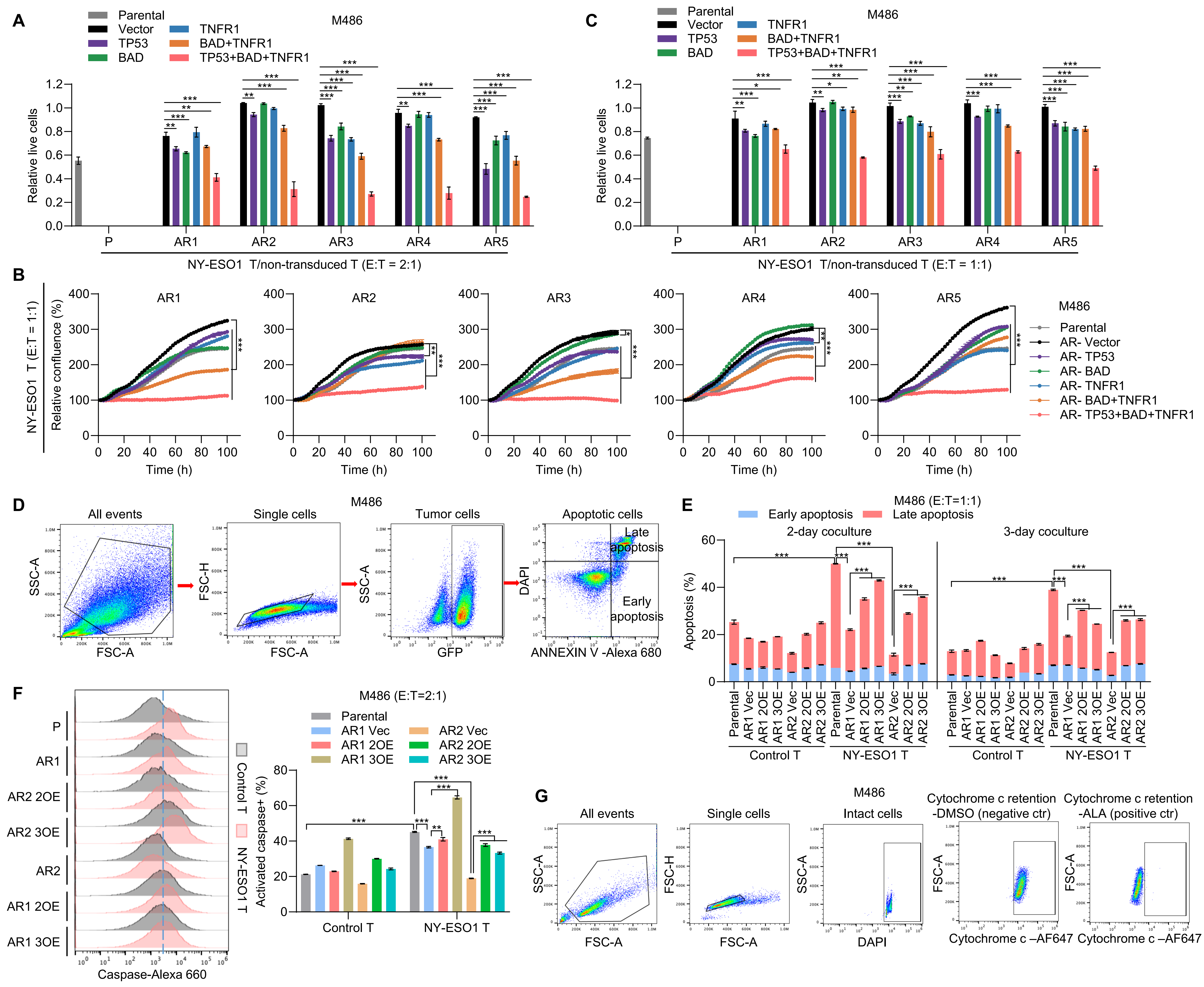

Figure S6. Perturbation studies of in vitro human melanoma AR models, related to Figure 6
(A) Quantification of endpoint data (Figure 6D) showing the ratios of live cells (M486 P line and AR sublines ± overexpression of indicated genes) in cocultures with HLA-/antigen-specific T cells versus non-transduced (control) T cells.
(B and C) As in Figure 6D and Figure S6A, except at E:T ratio of 1:1.
(D) Example (M486) of flow cytometry gating strategy for early and late apoptosis detection in Figure 6E and Figure S6E.
(E) As in Figure 6E, except at E:T ratio of 1:1.
(F) Detection of pan-activated caspases in the M486 P line and indicated AR sublines with empty vector (Vec), stable two-gene (*BAD*, *TNFR1*) overexpression (2OE), or stable three-gene (*P53*, *BAD*, *TNFR1*) overexpression (3OE), after cocultures for two days with non-TCR-transduced (control) or HLA-/antigen-specific TCR-transduced primary T cells (E:T ratio of 2:1).
(G) Example of flow cytometry gating strategy for detecting cytochrome c upon treatment with BH3-mimetic peptides. ALA, alamethicin, used as a positive control treatment to release cytochrome c.
Mean (triplicates) ± SEM; *P* values, one-way ANOVA followed by Tukey's multiple comparison test (A, C, E, F) or two-way ANOVA test (B).

Figure S7

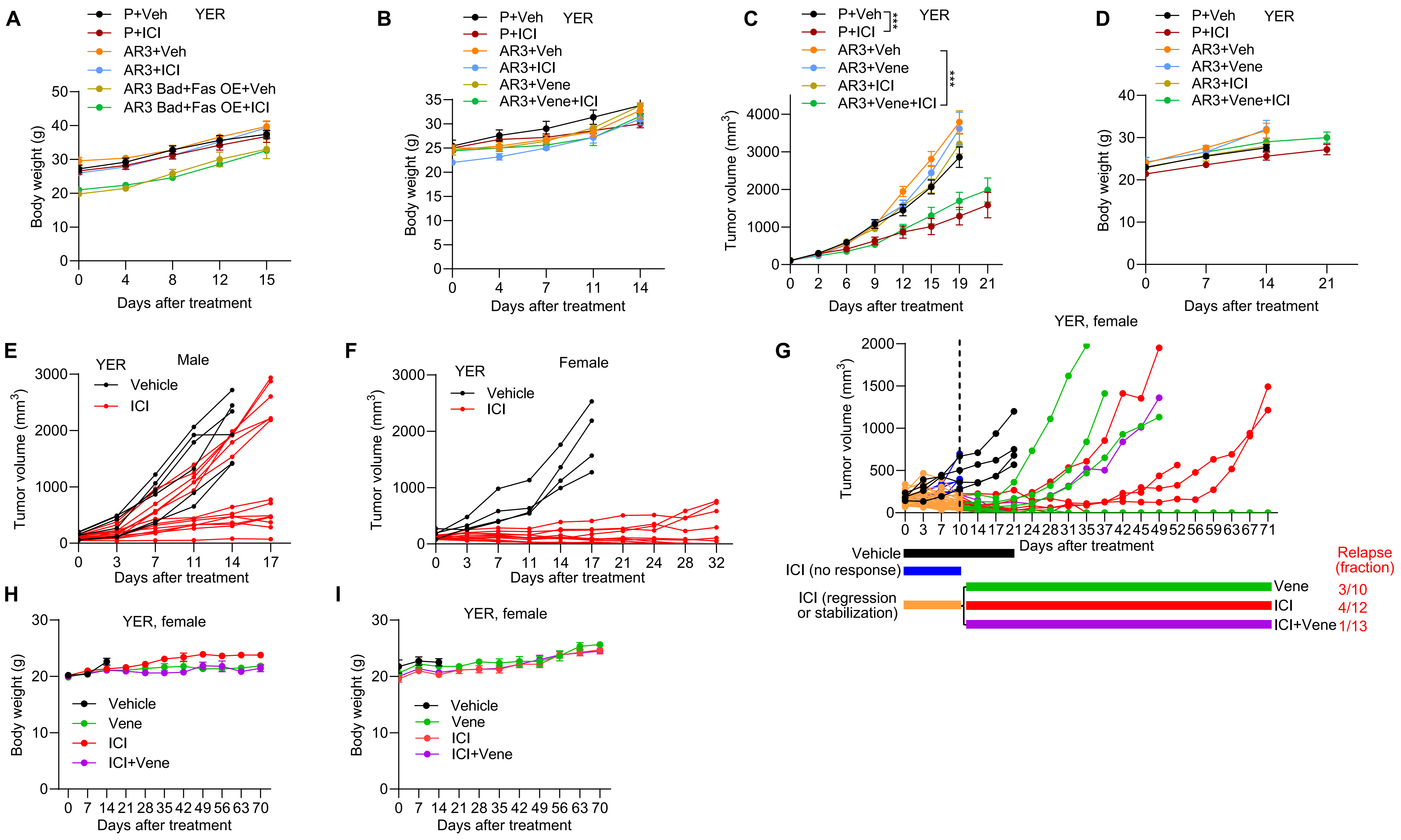

Figure S7. Perturbation studies of in vivo murine melanoma AR models, related to Figure 7
(A and B) Body weights of mice in Figure 7C and 7D (*n* = 5 mice/group).
(C and D) As in Figure 7D and Figure S7B, except an independent experiment. Mean ± SEM; *P* values, two-way ANOVA test (C).
(E and F) Growth curves of individual YER tumors in male (E) or female (F) UCLA C57BL/6ROC mice treated with vehicle or ICIs (anti-PD-1+anti-CTLA-4).
(G) As in Figure 7G, except an independent experiment (*n* = 52 tumors). *P* values, Fisher's exact test.
(H and I) Body weights of mice in Figure 7G and Figure S7G.
